# Broad-Spectrum Antimicrobial ZnMintPc Encapsulated in Magnetic-Nanocomposites with Graphene Oxide/MWCNTs Based on Bimodal Action of Photodynamic and Photothermal Effects

**DOI:** 10.3390/pharmaceutics14040705

**Published:** 2022-03-26

**Authors:** Coralia Fabiola Cuadrado, Antonio Díaz-Barrios, Kleber Orlando Campaña, Eric Cardona Romani, Francisco Quiroz, Stefania Nardecchia, Alexis Debut, Karla Vizuete, Dario Niebieskikwiat, Camilo Ernesto Ávila, Mateo Alejandro Salazar, Cristina Garzón-Romero, Ailín Blasco-Zúñiga, Miryan Rosita Rivera, María Paulina Romero

**Affiliations:** 1 Laboratorio de Nuevos Materiales, Departamento de Materiales, Facultad de Ingeniería Mecánica, Escuela Politécnica Nacional, Quito 170525, Ecuador; kleber.campana@epn.edu.ec (K.O.C.); maria.romerom@epn.edu.ec (M.P.R.); 2 School of Chemical Sciences and Engineering, Yachay Tech University, Urcuquí 100119, Ecuador; adiaz@yachaytech.edu.ec; 3 Instituto SENAI de Inovação, Serviço Nacional de Aprendizagem Industrial (Firjan SENAI), Rio de Janeiro 999074, Brazil; eromani@firjan.com.br; 4 Departamento de Ciencia de Alimentos y Biotecnología DECAB, Escuela Politécnica Nacional, Quito 170525, Ecuador; francisco.quiroz@epn.edu.ec; 5 Magnetic Soft Matter Group, Department of Applied Physics, Faculty of Sciences, University of Granada, 18071 Granada, Spain; stefania@ugr.es; 6 Centro de Nanociencia y Nanotecnología, Universidad de Las Fuerzas Armadas ESPE, Sangolquí 171103, Ecuador; apdebut@espe.edu.ec (A.D.); ksvizuete@gmail.com (K.V.); 7 Departamento de Física, Colegio de Ciencias e Ingenierías, Universidad San Francisco de Quito, Quito 170901, Ecuador; dniebieskikwiat@usfq.edu.ec; 8 Laboratorio de Investigación en Citogenética y Biomoléculas de Anfibios (LICBA), Centro de Investigación para la Salud en América Latina—CISeAL, Facultad de Ciencias Exactas y Naturales, Pontificia Universidad Católica del Ecuador, Quito 170143, Ecuador; camoavila@gmail.com (C.E.Á.); massuno@hotmail.com (M.A.S.); ccgarzonrom@outlook.com (C.G.-R.); ailin.blasco.z@gmail.com (A.B.-Z.)

**Keywords:** antimicrobial nanomaterials, carbon nanotubes, graphene, magnetic nanoparticles, hydrogel, photodynamic therapy, photothermal therapy, nanocarrier

## Abstract

Microbial diseases have been declared one of the main threats to humanity, which is why, in recent years, great interest has been generated in the development of nanocomposites with antimicrobial capacity. The present work studied two magnetic nanocomposites based on graphene oxide (GO) and multiwall carbon nanotubes (MWCNTs). The synthesis of these magnetic nanocomposites consisted of three phases: first, the synthesis of iron magnetic nanoparticles (MNPs), second, the adsorption of the photosensitizer menthol-Zinc phthalocyanine (ZnMintPc) into MWCNTs and GO, and the third phase, encapsulation in poly (N-vinylcaprolactam-co-poly(ethylene glycol diacrylate)) poly (VCL-co-PEGDA) polymer VCL/PEGDA a biocompatible hydrogel, to obtain the magnetic nanocomposites VCL/PEGDA-MNPs-MWCNTs-ZnMintPc and VCL/PEGDA-MNPs-GO-ZnMintPc. In vitro studies were carried out using *Escherichia coli* and *Staphylococcus aureus* bacteria and the *Candida albicans* yeast based on the Photodynamic/Photothermal (PTT/PDT) effect. This research describes the nanocomposites’ optical, morphological, magnetic, and photophysical characteristics and their application as antimicrobial agents. The antimicrobial effect of magnetics nanocomposites was evaluated based on the PDT/PTT effect. For this purpose, doses of 65 mW·cm^−2^ with 630 nm light were used. The VCL/PEGDA-MNPs-GO-ZnMintPc nanocomposite eliminated *E. coli* and *S. aureus* colonies, while the VCL/PEGDA-MNPs-MWCNTs-ZnMintPc nanocomposite was able to kill the three types of microorganisms. Consequently, the latter is considered a broad-spectrum antimicrobial agent in PDT and PTT.

## 1. Introduction

In recent years, the study of microorganisms has increased substantially due to their ability to spread rapidly and adapt to different environments. For this reason, the World Health Organization has declared microbial diseases as one of the main threats for humanity [1]. Currently, there are antibiotic therapies that help fight infections caused by microorganisms such as *Klebsiella pneumoniae*, *Escherichia coli*, *Staphylococcus aureus* and *Neisseria gonorrhoeae*, among others. However, these have been losing their efficacy, which is why the development of new treatments is necessary [2]. The generation of new nanocomposites that help eliminate microorganisms provides a new alternative for the fight against infections caused by these pathogens [3,4].

Nanotechnology for the control of infectious diseases includes several strategies, such as the use of metal oxides for the generation of reactive oxygen species (ROS), nanocomposites capable of damaging membrane integrity or causing physical damage to the bacterial wall [5], inhibiting DNA replication and adenosine triphosphate (ATP) production in cells [6], use of graphene-family materials for the microbial elimination [7,8,9,10], and others. In this regard, Romero et al., 2020 showed the ability of GO as an antimicrobial agent to eliminate *E. coli* and *S. aureus* bacteria through the combination of Photodynamic Therapy (PDT) and Photothermic Therapy (PTT) [11]. Mei et al., 2021, synthesized a ZnPc-TEGMME@GO nanocomposite, which has a thickness between 1.47 and 2.61 nm, using concentrations ≥25 μg·mL^−1^ of the nanocomposite and irradiation with dual light of 450 nm and 680 nm for 10 min. The nanocomposite increases its temperature to 100 °C and rapidly promotes singlet oxygen generation, causing cuts in the membranes of bacterial cells and, consequently, the death of Gram-positive and Gram-negative bacteria [12]. Yang et al., 2018, obtained the nonchemotherapeutic nanoagent Fe_3_O_4_-CNT-PNIPAM with a diameter of around 200 to 400 nm, which at a concentration of 0.1 mg·mL^−1^, and under 808 nm laser irradiation, 3 W·cm^−2^, for 5 min is capable of killing *S. aureus* and *E. coli* by PTT [13].

Photodynamic Therapy (PDT) is well known and studied for its use in cancer treatments, producing minimum side effects compared to other therapies used for cancer. PDT is an attractive operating modality based on the interaction of light with a photosensitizer (PS) and oxygen [14,15], thus producing ROS and free radicals capable of causing cell and microorganism death with high cytotoxicity [16,17,18,19]. This therapy has been used to treat skin diseases and cancers such as prostate, neck, lung, breasts and bladder cancers [20,21,22,23].

PS generally are classified by their activation wavelength, duration, and tissue penetration. One of the most widely used PS in PDT is the second generation PS, due to its photophysical and photochemical properties. Within this group are phthalocyanines, chlorins, and benzoporphyrins [24,25]. Phthalocyanines (Pcs) are hydrophobic compounds whose activation wavelengths of 650 and 700 nm allow deep tissue penetration. Pc reaches a high concentration in tumor tissue after 1 to 3 h of administration, and they are generally used to treat skin and subcutaneous lesions [26].

Zinc Menthol-Phthalocyanine (ZnMintPc) is a hydrophobic drug derived from phthalocyanine, whose structure is based on porphyrins but with a central Zinc atom with four methoxy groups around it that allow PS to be soluble in certain organic solvents [27]. The use of ZnMintPc has been limited due to its hydrophobic nature. When encapsulated by hydrogels, Pcs is soluble in aqueous media [28,29,30]. Pcs can also be combined with nanoparticles (Np) to create hybrid nanostructures that increase the quantum yield of singlet oxygen, cell uptake, and their therapeutic effectiveness [31,32].

Due to the hydrophobic nature of PS, several types of nanocarriers have been studied that prevent them from adding to each other and losing their physicochemical characteristics [20,33,34]. Among these nanocarriers are carbon nanostructures such as carbon nanotubes [35,36,37], graphene [33] and fullerenes [20,38,39], among others. These functionalized nanocarriers have excellent optical and mechanical properties. These compounds have cytotoxicity in biological systems, depending on their concentration, size, surface property and functional groups [40]. The surface modification of these nanocarriers with biomolecules such as polyethyleneimine, polyethylene glycol, and human proteins, improves the cytotoxicity and biocompatibility for biological applications [41], in addition to having other applications such as immunotherapy, imaging, and the development of vaccines and antimicrobial agents [42,43,44,45,46].

Photothermal treatment (PTT) is a type of phototherapy that works by turning light energy into heat through the use of photothermal agents (PTAs) [47]. There has been great interest in applying PTT to eliminate pathogenic bacteria in recent years. PTAs, when irradiated with near-infrared (NIR) light, generate much heat, causing protein denaturation, rupture of the bacterial cell membrane, and death of microorganisms [13,48,49]. An ideal PTA must meet specific requirements, such as high photothermal conversion efficiency, biocompatibility, ease of synthesis, photostability, and rapid elimination [50,51]. The best known PTAs are carbon-based nanomaterials, conjugated polymer-based nanomaterials, inorganic, and small molecule-based nanomaterials [48,52].

PTT/PTT synergies are considered among the most effective methods for microbe killing due to high specificity, minimal invasiveness, low risk of developing drug resistance, and selectivity [53,54]. These phototherapy techniques have a dual mode of action in which the PTT facilitates the absorption of PS because the increase in temperature will causes an increase in the permeability of the microorganism. At the same time, by PDT, the PS produces ROS, which can destroy proteins, lipids, and microbial DNA, causing the death of the bacteria, and increasing the effectiveness of the PTT since they are capable of reducing the heat resistance of bacteria [12,48].

CNTs were discovered by Iijima in 1991 [55] and consist of quasi-one-dimensional structures formed by several layers of graphene rolled up coaxially to form tubes. They are classified into two types: those with a single layer known as single-wall carbon nanotubes (SWCNTs), and those with several layers known as multiwall carbon nanotubes (MWCNTs) [56,57]. CNTs possess excellent optical, electrical, thermal, physical, and kinetic properties, and excellent cell permeability [58,59]. For this reason, there is much interest in their application as drug transport systems, electrochemical biosensors, and biological markers, among others [60,61].

CNTs have been used as biosensors, biomarkers, and drug transporters with a high carrying capacity [62,63]. Hybrid compounds have been created to improve the effectiveness of their action. Proteins, polymers, cell recognition ligands, nanoparticles, hydrophilic coatings have been incorporated into these structures, which provide them with new functions such as cell recognition, and controlled drug release, avoiding aggregations in aqueous media during targeted transport [64,65,66].

Graphene is a two-dimensional nanostructure with sp^2^ hybridization and strongly cohesive carbons, which gives the structure excellent optical, electronic, mechanical, and chemical properties, which vary depending on its lateral size [67]. Graphene is a material with very high resistance and hardness. It is light and has low toxicity and increased flexibility, making it an innovative material in construction, technology, medicine, and other industries [43,68,69]. Because of its excellent physicochemical characteristics, graphene has been used in the biomedical field to make biosensors, drug delivery systems, antibacterial agents for early detection of cancer, gene therapy, and for cancer cell imaging/mapping, among other uses [70].

In recent years, great interest has arisen in the synthesis of magnetic nanoparticles due to their unique physicochemical properties. They can be used in PPT/PDT [71], drug delivery, theranostics, and others [72,73,74]. Magnetic nanoparticles (MNPs), such as magnetite and hematite obtained from iron oxides, are widely used in biomedicine because they are biocompatible and have no cytotoxic effects at concentrations below 100 μg·mL^−1^. For breast, glia, and normal cells with cancer [75], at higher concentrations, an interaction between cell membrane phospholipids and iron nanoparticles occurs, resulting in membrane failure [76,77,78]. MNPs can be anchored to carbon structures to obtain drug nanocarriers and directed by an external magnetic field [79,80].

VCL/PEGDA is a biocompatible hydrogel that can be obtained by emulsion polymerization. This hydrogel is very promising since it responds to physiological changes in the temperature of the human body [81,82]. Therefore, it can be used as a drug delivery system to encapsulate hydrophobic and hydrophilic agents. A study carried out by Romero et al., 2021 showed the sustained release capacity of the drug colchicine encapsulated in VCL/PEGDA [83].

PS must meet specific requirements for PDT strategy. They must be selective, disperse well in tissue, and their photostability time must be adequate for the treatment. Due to these needs, we developed two magnetic nanocarriers based on MWCNTs and GO materials in this work. Both nanocarriers are decorated with Fe-MNPs, giving the compounds superparamagnetic properties. These nanocarriers were functionalized with PS ZnMintPc, a drug used in Photodynamic Therapy. A VCL/PEGDA hydrogel was used to help with dispersion of the hydrophobic compounds ZnMintPc and MWCNTs in aqueous media by providing them with a lipophilic envelope. Finally, the antimicrobial effect of these nanocomposites was evaluated to eliminate the bacteria *S. aureus*, *E. coli*, *C. albicans* using the PDT/PTT strategy.

## 2. Materials and Methods

### 2.1. Materials

For the synthesis and functionalization process, MWCNTs and GO were provided by the Van de Graff Laboratory, Department of Physics PUC-RIO, Rio de Janeiro, Brazil. Zinc Menthol-Phthalocyanine was provided by the Federal University of São Carlos, São Carlos, Brazil; Fe(SO_4_), H_2_SO_4_·7H_2_O from MERCK, Rio de Janeiro, Brazil; NH_4_OH, 14.8N and N,N Dimethylformamide from Fisher Scientific, Princeton, NJ, USA; Fe_2_(SO_4_)_3_·H_2_O from Fisher Scientific. HNO_3_ from the Fermont, Monterrey, Mexico; saline solution Fisiol UB (pH = 7) from Lamosan, Quito, Ecuador and Tween80 from La casa del Químico, Quito, Ecuador.

For VCL/PEGDA hydrogel synthesis by emulsion polymerization, the following reagents were used. N-vinylcaprolactam (VCL; Sigma Aldrich, Darmstadt, Germany, 98%), and Poly(ethylene glycol) diacrylate (PEGDA; Sigma Aldrich, Mn 250), the initiator ammonium persulfate (APS; FMC Corporation, Philadelphia, PA, USA, >99%), the emulsifier sodium dodecyl sulfate (SDS; STEOL^®^CS-230 Stepan, Northbrook, IL, USA) and a buffer of sodium hydrogen carbonate (Sigma Aldrich, ≥99.7%) were used as provided.

Characterizations of MNPs-MWCNTs and MNPs-GO were carried out by FT-IR spectroscopy analysis in a JASCO FT/IR-4100 spectrometer, JASCO International Co., Ltd., Tokyo, Japan. ES (wavenumber range 7800 to 350 cm^−1^, resolution of 0.7 cm^−1^) and Raman spectroscopy analysis in a HORIBA Raman spectrometer LabRAM HR Evolution (Horiba, Kyoto, Japan), where the samples were excited with a 2.33 eV (532 nm).

Magnetization (*M*) measurements were carried out using a Quantum Design Versalab VSM, Quantum Design, Darmstadt, Germany. FR, magnetometer in the temperature range between −210 and +60 °C with applied magnetic fields, μ_0_H, up to 3 T.

Stability over time of the PS magnetic nanocomposites was characterized by UV-VIS spectroscopy analysis at a wavelength range of 280 to 780 nm (resolution better than 1.8 Å), using a UV-VIS Spectrophotometer model Evolution 220 from Thermo Fisher Scientific, Waltham, MA, USA. DPBF photobleaching and thermic studies were carried out with homemade equipment using an LED red light at 635 nm, 65.5 mW·cm^−2^. A DPBF photobleaching study was characterized in a UV-VIS Specord 210 Plus. XRD analysis was performed on a PANalytical brand EMPYREAN Diffractometer (Malvern Panalytical, Malvern, UK) operating in a θ–2θ configuration (Bragg-Brentano geometry) equipped with a copper X-ray tube (Kα radiation λ = 1.54056 Å) at 45 kV and 40 mA.

### 2.2. Methods

#### 2.2.1. Morphological Studies

Scanning Electron Microscopy (SEM) and Energy Dispersive X-ray Spectroscopy (EDS) evaluated morphology and semiquantitative elemental composition. For this, an aliquot of the sample was fixed to an aluminum sample holder using a double layer of carbon tape. The analyses were carried out on an SEM Tescan Mira 3 (Tescan, Brno, Czech Republic). equipped with a Schottky field emitter (JEOL Ltd., Tokyo, Japan). EDS was performed in the SEM chamber using a Bruker, X-Flash 6|30 detector (Bruker, Billerica, MA, USA) with a resolution of 123 eV at Mn Kα. Tapping-mode atomic force microscopy was used to determine the shape and thickness of GO (Bruker Dimension Icon AFM).

For Transmission Electron Microscopy (TEM), the samples were dispersed in a BRANSON 1510 ultrasonic (Transcat, Rochester, NY, USA) bath for 30 min. Next, approximately 5 µL of the sample was placed on a TEM grid (formvar/carbon, 300 mesh), and the solvent was removed with filter paper. The samples were observed in a TEM FEI, Tecnai G2 Spirit Twin (ELECMI, Zaragoza, Spain) equipped with an Eagle 4k HR camera (Tronic Extreme, Coventry, UK) at 80 kV.

For scanning transmission electron microscopy (STEM), 5 µL of the sample was placed on a TEM grid (formvar/carbon, 300 mesh), and the solvent was removed with filter paper. Staining was performed with 1% Phosphotungstic Acid for 1 s for *S. aureus* and 1 min for *E. coli*, the solvent was removed, and the sample was observed in an SEM Tescan, Mira 3 in transmission mode.

#### 2.2.2. Synthesis of Graphene Oxide

GO was prepared according to Hummers’ method [84]. Graphite powder with 99% purity was used for the synthesis of GO. Chemical products, including NaNO_3_, KMnO_4_, H_2_SO_4_, and aqueous solutions of H_2_O_2_, and HCl, were purchased from Sigma Aldrich. GO powder was obtained after lyophilization of the suspected GO in deionized water.

#### 2.2.3. Purification of MWCNTs

The purification of MWCNTs was carried out using an acid attack. A 5.3 mg sample of MWCNTs was dispersed with 5% Tween 80 in 10 mL of distilled water stirred at 200 rpm for 24 h to ensure that the MWCNTs were well dispersed. Then, in a solution of 4 mL of HNO_3_ and 12 mL of H_2_SO_4_ (1:3 ratio), the solution of MWCNTs previously treated was allowed to cool, and then stirred magnetically for 3 h. Several washes of the MWCNTs were performed using 0.22 µm pore micropore filtration until a neutral pH was obtained.

#### 2.2.4. Synthesis of MNPs-MWCNTs and MNPs-GO

In 18 mL of pure water, the following were dispersed: purified MWCNTs, 225 mg of FeSO_4_, 450 mg of (Fe_2_ (SO_4_)_3_), and 5% Tween 80. The sample was placed in a magnetic stirrer for 3 h, then was added carefully to 150 mL of NH_4_OH. The mixture was exposed to magnetic stirring in an inert atmosphere for 1 h at 200 rpm. Finally, several magnetic purifications of the MNPs-MWCNTs nanocarrier were carried out, until the pH was neutralized, and allowed to dry.

The same process was used to synthesize of MNPs-GO, using 5.3 mg of GO instead of MWCNTs.

As a control sample for the magnetic measurements, free-standing iron nanoparticles were prepared using the same co-precipitation method but without GO or MWCNTs.

#### 2.2.5. Synthesis of Polyethylene Glycol Diacrylate-Vinylcaprolactam (VCL/PEGDA)

Hydrogel synthesis was carried out by emulsion polymerization of 2 g of VCL, 0.08 g of PEGDA, STEOL CS-230, and 0.08 of NaHCO_3_ dispersed in 235 mL deionized water. The mixture was slowly placed in the chemical reactor with stirring at 350 rpm and a temperature of 70 °C, maintaining a stream of Nitrogen for one hour. Then, the initiator (0.03 g of Ammonium Persulfate dissolved in 15 mL of distilled water) was added to the solution, and the reaction was kept at 70 °C for 7 h. After this time, the mixture was allowed to cool under stirring at 200 rpm and 25 °C to avoid aggregation for 12 h. Finally, the hydrogel was dialyzed against DDI water to remove impurities and unreacted reagents [83].

#### 2.2.6. Functionalization of MNPs-MWCNTs; MNPs-GO with ZnMintPc in the Presence of VCL/PEGDA

In 10 mL of pure water, 2 mg of MNPs-MWCNTs were dispersed with 5% Tween, and the sample stirred magnetically for 24 h at 200 rpm, then sonicated for 30 min. A volume of 10 mL of VCL/PEGDA was added to the mixture, and it was stirred magnetically for 4 h at 200 rpm.

To 20 mL of the VCL/PEGDA-MNPs-MWCNTs solution, 0.67 mL of ZnMintPc solution (0.25 mM) were added, after which the mixture was sonicated for 4 h at 250 rpm. The solution was covered with aluminum foil to avoid ZnMintPc photodegradation.

The same process was carried out with MNPs-GO, obtaining the VCL/PEGDA-MNPs-GO nanocomposite.

#### 2.2.7. Quenching Experiment of 1,3-Diphenyl Isobenzofuran

The fluorescence and absorption characteristics of 1,3-diphenylisobenzofuran (DPBF), a singlet oxygen trapping chemical, are widely employed to detect and quantify singlet oxygen [85]. This agent has an absorption range between 410–420 nm, emitting blue fluorescence. When DPBF interacts with oxygen, it produces o-dibenzoylbenzene, without absorbing visible light. The amount of ^1^O_2_ produced is shown by a reduction in DPBF absorbance [11]. A sample of 5 mg of DPBF was dispersed in 1 mL of DMF to distribute in the solutions with nanocomposites later.

For this experiment, a UV-VIS SPECORD 210 Plus spectrophotometer (resolution of 2.3–2.5 nm) in a range of 300 to 800 nm was used. A reference solution was prepared with 10 mL of deionized water and 10% Tween 80. Then, in 3 mL of this reference solution, 100 µL of VCL/PEGDA-MNPs-GO (0.1 mg/mL) was added. The same process was repeated with the nanocomposites VCL/PEGDA-MNPs-MWCNTs (0.1 mg/mL), VCL/PEGDA-MNPs-MWCNTs-ZnMintPc (0.1 mg/mL) and VCL/PEGDA-MNPs-GO-ZnMintPc (0.1 mg/mL). After that, 20 µL of the DPBF (18.5 mM) solution was placed in each of them, and they were irradiated at different times while observing a decrease in absorbance of 418 nm due to DPBF.

#### 2.2.8. Thermal Studies

Thermal studies were carried out by irradiating, in deionized water, VCL/PEGDA, MNPs, GO, MWCNTs, VCL/PEGDA-MNPs-GO, and VCL/PEGDA-MNPs-MWCNTs, with a red light of 630 nm and 65.5 mW·cm^−2^ and taking the temperature every 5 to 10 min until it did not change.

#### 2.2.9. Antimicrobial Studies

The antimicrobial study used cryovials with the microorganisms *Staphylococcus aureus* ATCC: 25923, *Escherichia coli* ATCC: 25922, and *Candida albicans* ATCC: 10231 as test subjects. Cryovials were thawed at room temperature, and the contents were inoculated in Mueller-Hinton broth (Difco^TM^). The culture medium was incubated overnight at 37 °C. After this period, the absorbance of each medium was determined by spectrophotometry (Thermo Scientific ^TM^ Orion ^TM^ AquaMate 8000 UV-Vis, Thermo Fisher Scientific, Waltham, MA, USA) and diluted in Mueller-Hinton broth to reach the concentration established for the bioassays: 10^7^ CFUs mL^−1^ for *E. coli*, 10^6^ CFUs mL^−1^ for *S. aureus*, and 10^5^ CFUs mL^−1^ for *C. albicans*.

Upon reaching the indicated concentration, each medium was dispensed into microtubes: six aliquots of 1 mL per magnetic nanocomposites (VCL/PEGDA-MNPs-GO-ZnMintPc, VCL/PEGDA-MNPs-MWCNTs-ZnMintPc, VCL/PEGDA-MNPs-GO, and VCL/PEGDA-MNPs-MWCNTs) and six aliquots of 1 mL as control. Half of the aliquots were used to evaluate the activity of the magnetic nanocomposites under light irradiation, while the other half was not exposed to these conditions.

The microtubes were centrifuged at 3000 rpm for 10 min. The supernatant was discarded, and 1 mL of PBS was added to wash the cells and remove the remains of the culture medium. The pellet was resuspended and recentrifuged under the same conditions. PBS was discarded and 1 mL of the compound was dispensed into each microtube. In the control microtubes, the cells were resuspended again with PBS. Each tube was vortexed to dissolve the pellet again and incubated at 37 °C in the dark for 45 min.

At the end of the incubation period, three aliquots of each compound and three controls were subjected to light irradiation using red light of 630 nm, 65.5 mW·cm^−2^. The remaining tubes were not irradiated.

Serial dilutions of each aliquot were made in PBS. A 4 µL aliquot of each dilution was inoculated into an 8-part Petri dish with Mueller-Hinton agar (Difco^TM^). Each inoculum was streaked for colony isolation. Petri dishes were incubated for 24 h at 37 °C, and after this period the number of colonies was counted per dilution.

## 3. Results

### 3.1. Composition and Structure Characterization of Magnetic Nanocomposites

**Figure 1:**
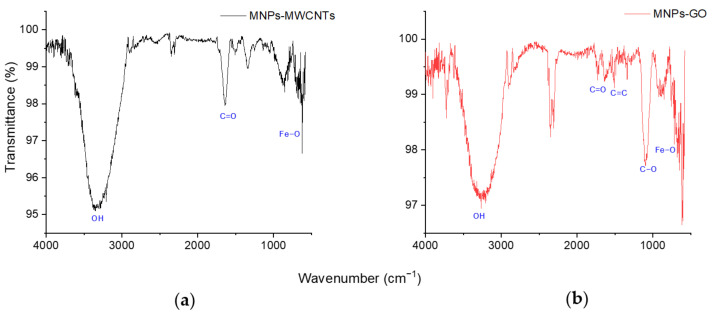
FT-IR spectra: (**a**) MNPs-MWCNTs; (**b**) MNPs-GO.

FT-IR spectroscopy was performed on the magnetic nanocomposites to study the difference in oxygen-related functional groups (Figure 1). Figure 1a shows the FT-IR spectra of MNPs-MWCNTs, which presents characteristic bands. As the literature indicates, the band at 3373 cm^−1^ is attributed to the vibration of the hydroxyl group (OH), the 1636 cm^−1^ band corresponding to the FeOO and shows the successful decoration of MWCNTs with iron nanoparticles through hydrogen bonds [86,87,88]. Other bands appear in the range 1400–1730 cm^−1^, corresponding to the OH–C=O, –COO, –COOH groups added due to acid treatment and functionalization with MNPs. Finally, the characteristic bands of the MNPs appear at 709 and 623 cm^−1^, which indicate the stretching vibration of Fe–O–Fe characteristic of Fe-MNPs, which agrees with Abrinaei, Kimiagar and Zolghadr 2019 [89].

Figure 1b presents the FT-IR spectra of the MNPs-GO nanocarrier where characteristic bands of GO are identified. There is a band around 3327 cm^−1^ corresponding to the bending and vibration of the OH stretching of the C-groups, the band at 1636 cm^−1^ corresponds to the vibration of the C=C, the bands around 800 cm^−1^ and 1269 cm^−1^ represent the epoxy group, and bands at 1342 cm^−1^, 1247 cm^−1^, 1049 cm^−1^ show the stretching vibration of the carboxy groups C−O, C−C−O, and alkoxy C−O, respectively; as shown in the work of Al-Ruqeishi et al., 2020 [90].

**Figure 2:**
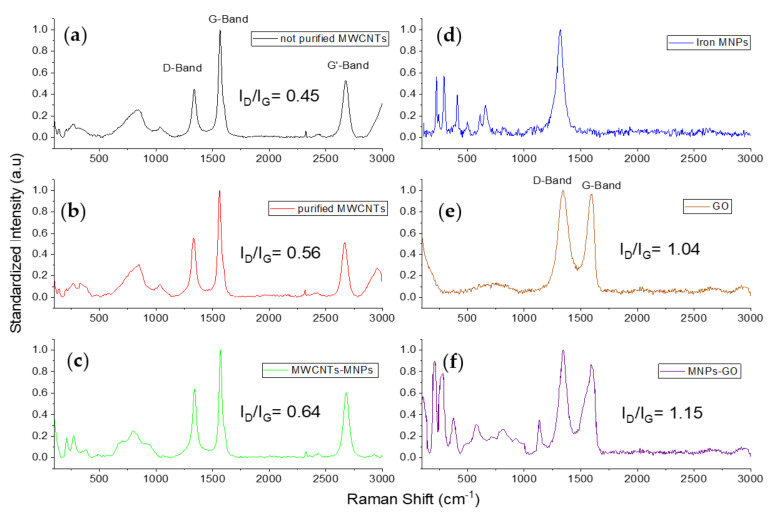
Raman Spectra: (**a**) non-purified MWCNTs, (**b**) purified MWCNTs, (**c**) MNPs-MWCNTs; (**d**) Fe-MNPs; (**e**) GO; (**f**) MNPs-GO; E_láser_ = 2.33 eV, λ = 532 nm.

Figure 2a shows the Raman spectra of non-purified MWCNTs (Figure 2a), purified MWCNTs (Figure 2b), and MNPs-MWCNTs (Figure 2c). These spectra show characteristic bands of the MWCNTs: band D or band of defects and the first and second-order bands G and 2D, respectively. The D-band is at 1337 cm^−1^ and indicates the presence of defects in the graphite, resulting from the presence of multiple carbon sheets that are not directly aligned sheet to sheet, which induces a loss of translational symmetry in the two-dimensional network. Due to the same effect, a secondary phonon is produced that gives rise to the presence of the G-band at 1566 cm^−1^. The fundamental band G is a tangential elongation band attributed to the in-plane vibration of the C-C bond, is typical of carbon-derived materials, and is consistent with reports in the literature [91,92,93,94].

Figure 2d shows the Raman spectra of Iron-MNPs with its characteristic bands, two A_1g_ modes at 226, 502 cm^−1^, five E_g_ modes at 248, 291, 300, 407 and 615 cm^−1^, and the characteristic two-magnon scattering band at 1320 cm^−1^, which agree with the results presented by Soler and Qu 2012 [95]. Some of these bands can be observed in the spectra shown in Figure 2c,f.

Figure 2a,b show Raman spectra of unpurified MWCNTs and purified MWCNTs. The $\frac{I_{D}}{I_{G}}$ ratio of the purified MWCNTs is higher than that of unpurified MWCNTs because treatment with acids causes some bonds to break and form functional groups, generating defects in the structure of MWCNTs.

In the Raman spectrum of MNPs-MWCNTs (Figure 2c), the increase in the $\frac{I_{D}}{I_{G}}$ ratio is due to charge transfer effects between MNPs and MWCNTs; the result of the functionalization is a structure of MWCNTs with defects. In addition, extra bands that correspond to the MNPs are observed.

Figure 2e shows the characteristic bands of GO, an intense D band at 1340 cm^−1^, a G-phase vibration band at 1589 cm^−1,^ and the D + D’ band located around 2900 cm^−1,^ which is activated by defects and appears with a combination of phonons with different linear moments around points K and Γ in the Brilloüin zone and agrees with Cançado et al., 2011, and Muhammad Hafiz et al., 2014 [96,97]. The ratio of the bands $\frac{I_{D}}{I_{G}}$ = 1.036 results from the degree of disorder in GO due to the presence of Carboxylic acid functional groups at its ends.

Figure 2f shows the spectra of the MNPs-GO nanocomposite where there is a shift of D band, since the nature of iron-MNPs when combined with carbon nanostructures affects the spectral amplification of the phonon peaks, agreeing with the information presented by Ramirez et al., 2017 and Satheesh et al., 2018 [98,99].

### 3.2. Magnetic Properties

**Figure 3:**
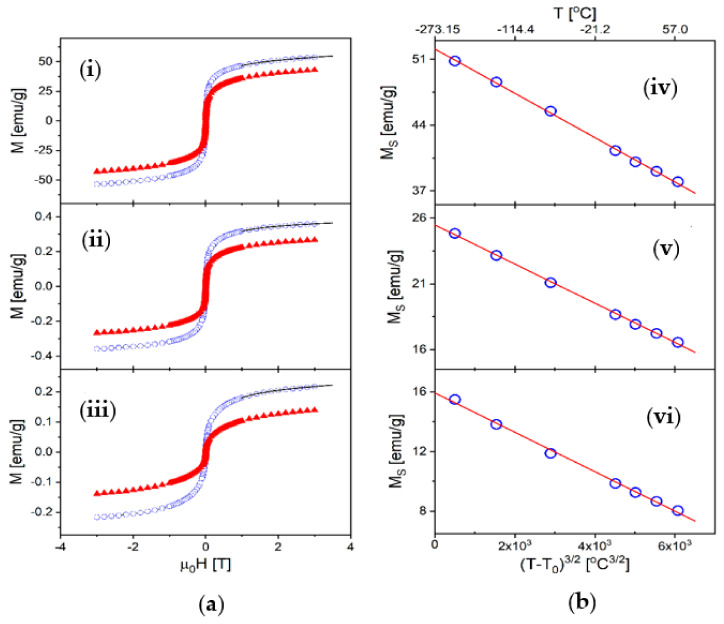
Magnetic properties: (**a**) Magnetization vs. applied magnetic field: (**i**) the free-standing nanoparticles, (**ii**) VCL-PEGDA-MNPs-MWCNTs, and (**iii**) VCL-PEGDA-MNPs-GO. The curves shown reflect measurements at two different temperatures, T = −210 °C (circles) and T = 20 °C (triangles). The solid lines are examples of the fit of the data using the law of approach to saturation (LAS) of Equation (1). (**b**) Saturation magnetization as a function of temperature: (**iv**) the free-standing nanoparticles, (**v**) VCL-PEGDA-MNPs-MWCNTs and (**vi**) VCL-PEGDA-MNPs-GO. The solid lines match the data showing that MS follows Bloch’s law given Equation (2).

We studied the magnetic properties of three samples: the nanocomposites VCL/PEGDA-MNPs-MWCNTs and VCL/PEGDA-MNPs-GO and, for comparison, the free-standing iron nanoparticles (MNPs). All the studied samples showed Fe nanoparticles’ typical ferromagnetic (FM) behavior, with a very small coercivity of less than 2 mT at room temperature (less than 5 mT at T = −210 °C). This small coercivity indicates that the MNPs were not directly attached to the carbon structures, since directly attached MNPs have large coercivities of hundreds of mT [100,101]. This was expected because the hydrogen bonds present between MNPs and carbon structures mentioned in the results of the FT-IR spectroscopy in Section 3.1. are weak and therefore generate a reduction of the Fe–C magnetic coupling. Figure 3a shows some representative *M* vs. *H* loops measured at two different temperatures, −210 °C and +20 °C, for the three samples. From these curves we obtained saturation magnetization (*M_S_*) as a function of temperature using the usual law of approach to saturation (LAS) [102,103,104] given by:

(1) $$M\left(H\right)=M_{S}\left(1-\frac{a}{H}-\frac{b}{H^{2}}\right)+\chi H,$$

In this equation, *a* and *b* are constants, and the last term accounts for the non-ferromagnetic contributions, such as the disordered shell of the nanoparticles. The latter is valid only at high fields, close to saturation; thus, we used it to fit our *M*(*H*) curves for applied fields between 1 and 3 T, as shown by the solid lines in Figure 3a for T = −210 °C. From these fits we obtained *M_S_* at several temperatures between −210 °C and +60 °C (see Figure 3b). In the case of the VCL-PEGDA-MNPs-MWCNTs and VCL-PEGDA-MNPs-GO samples, the magnetization value appeared substantially reduced since most of the mass (>98%) corresponds to the hydrogel. Therefore, to directly compare the measured magnetic moment with that of pure iron, the saturation magnetization value was corrected by the mass fraction of nanoparticles, x = 1.43% for the VCL-PEGDA-MNPs-MWCNTs and x = 1.37% for the VCL-PEGDA-MNPs-GO sample.

The saturation magnetization as a function of temperature is presented in Figure 3b, where we also show that, for all three samples, *M_S_* closely follows the well-known Bloch’s law for FM nanoparticles [105,106,107]:

(2) $$M_{S}\left(T\right)=M_{0}\times \left[1-B\;{\left(T-T_{0}\right)}^{\frac{3}{2}}\right],$$

where *M*_0_ is the saturation magnetization at the absolute zero degrees Kelvin (*T*_0_ = −273.15 °C), and *B* is the so-called spin-wave constant. The results for these magnetic parameters are presented in Table 1. The values obtained for *B* are very similar to those measured in other Fe-nanoparticles systems [104,105] (B ~ 10^−5^–10^−4^ °C^−3/2^) implying the existence of comparable thermally induced magnetic field excitations within the FM volume. On the other hand, our results show that the saturation magnetization is, in all cases, much smaller than that of pure iron, *M_Fe_* = 222 emu·g^−1^. However, in the case of the free-standing nanoparticles, *M_S_* remains larger than for the nanoparticles submerged within the hydrogel, indicating that the non-FM (disordered) shell increases in the presence of the gel medium. Moreover, the difference between the samples becomes even greater at room temperature, where the saturation magnetization decreases due to thermal effects (see Table 1). From this analysis, the effective FM volume (the size of the FM core of the nanoparticles) can be estimated by comparing *M*_0_ with *M_Fe_*, such that the fraction of the nanoparticle volume that remains, FM, can be estimated as:

(3) $$f=\frac{M_{0}}{M_{Fe}},$$

The very small FM volume fraction in the case of the VCL-PEGDA-MNPs-MWCNTs and VCL-PEGDA-MNPs-GO samples likely indicates that the presence of the hydrogel induces strong oxidation of the surface of the nanoparticles or some interdiffusion that corrodes the surface and reduces the magnetic moment. Moreover, the larger value of the spin-wave constant in these two samples is consistent with a more significant influence of surface effects [107], which produce a faster decrease in saturation magnetization when the temperature increases.

### 3.3. Optical Properties of Magnetic Nanocomposites

UV-VIS absorbance spectra and photoluminescence emission were obtained. In the Supplementary Material (Figure S1a,b,d), we show the ZnMintPc-DMF and the nanocomposites VCL/PEGDA-ZnMintPc and VCL/PEGDA-MNPs-MWCNTs-ZnMintPc, respectively. We observed the presence of bands at 354 nm (Band of B or Soret), 616 nm, and 684 nm (B and Q), characteristic of the absorption spectra of ZnMintPc.

Furthermore, it can be seen that as the concentration of ZnMintPc increases, the absorption of the B and Q bands increases proportionally without their dislocation. This allows us to conclude that the Beer-Lambert law is fulfilled and there is no drug aggregation, indicating that N,N-Dimetilformamida (DMF) is a suitable solvent for this PS [108,109]. Furthermore, it is also concluded that the VCL-PEGDA hydrogel disperses PS similarly to DMF. In the Supporting Information (Figure S1c,d), the UV-VIS spectra of nanocomposites based on MNPs-MWCNTs (VCL-PEGDA-MNPs-MWCNTs and VCL-PEGDA-MNPs-MWCNTs-ZnMintPc) are presented, showing the band corresponding to MWCNTS at 265 nm, which is mentioned in the research by Wang et al., 2012 [110].

Figure S2a in the Supporting Information shows UV-VIS spectra of MNPs-GO, where GO shows a π→π* transition of aromatic C–C bonds in the 230 nm band, which cannot be displayed, and a shoulder around 315 nm attributed to the *n*→π* transitions of C=O bonds, as in Bera et al., 2017 [111]. The last band has undergone a hypsochromic shift due to functionalization with both MWCNTs and GO in the presence of an organic solvent (ammonium hydroxide used for MNPs syntheses), as described in the literature [112,113].

The calibration curve shown in Figure S3a was obtained from the Q band at 684 nm related to ZnMintPc and described in Figures S1 and S2, which is in a region of the spectra of interest for treatment in PDT. It can be observed that the higher the concentration of ZnMintPc in the nanocomposites (VCL-PEGDA-ZnMintPc, VCL-PEGDA-MNPs-GO-ZnMintPc, and VCL- PEGDA-MNPs-MWCNTs-ZnMintPc), the greater the intensity of the 684 nm band; but when comparing with the ZnMintPc dispersed in DMF, the latter shows the highest intensity in relation to all nanocomposites. This indicates that the VCL-PEGDA hydrogel adequately disperses the hydrophobic ZnMintPc PS in an aqueous solution, as shown in the literature [114,115,116]. It can be seen that the 684 nm absorption band of ZnMintPc (0.52 µM) decreases as the number of functionalized components increases, as presented in Figure S3b. The percentage of decrease for VCL/PEGDA-MNPs-MWCNTs-ZnMintPc nanocomposites is 38.2%, and for VCL/PEGDA-MNPs-GO-ZnMintPc it is 35.54%.

**Figure 4:**
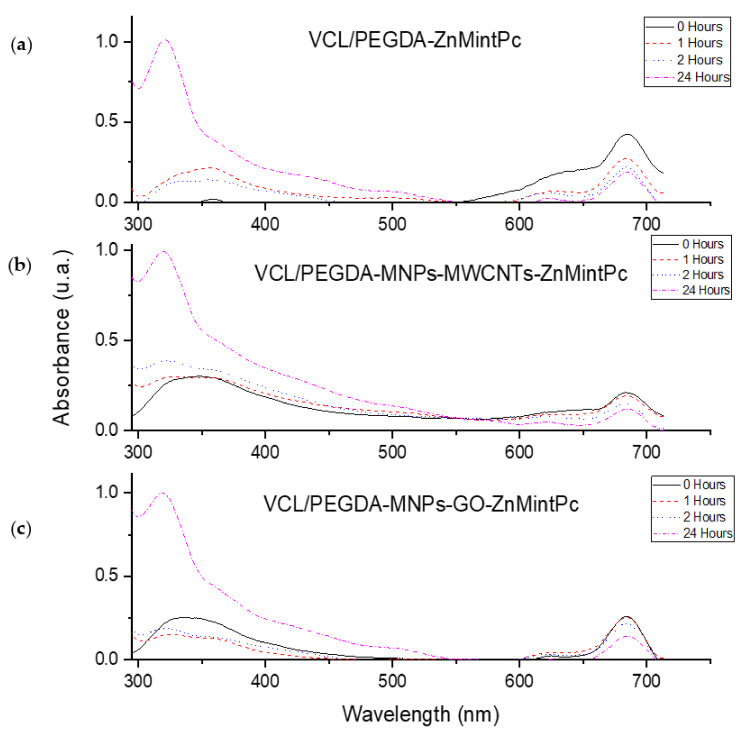
Stability curves over time for (**a**) VCL-PEGDA-ZnMintPc; (**b**) VCL/PEGDA-MNPs-MWCNTs-ZnMintPc and (**c**) VCL/PEGDA-MNPs-GO-ZnMintPc; ZnMintPc = 0.27 µM.

The temporal stability study of the VCL/PEGDA-ZnMintPc nanocomposites VCL/PEGDA-MNPs-MWCNTs-ZnMintPc and VCL/PEGDA-MNPs-GO-ZnMintPc, observed in Figure 4, was conducted at one, two and 24 h. In all three cases, as time passed, the intensity of the B band increased while the Q band decreased. In all three cases, as time passes, the intensity of the B band increased while the Q band decreased due to PS photobleaching without aggregation in 24 h.

**Figure 5:**
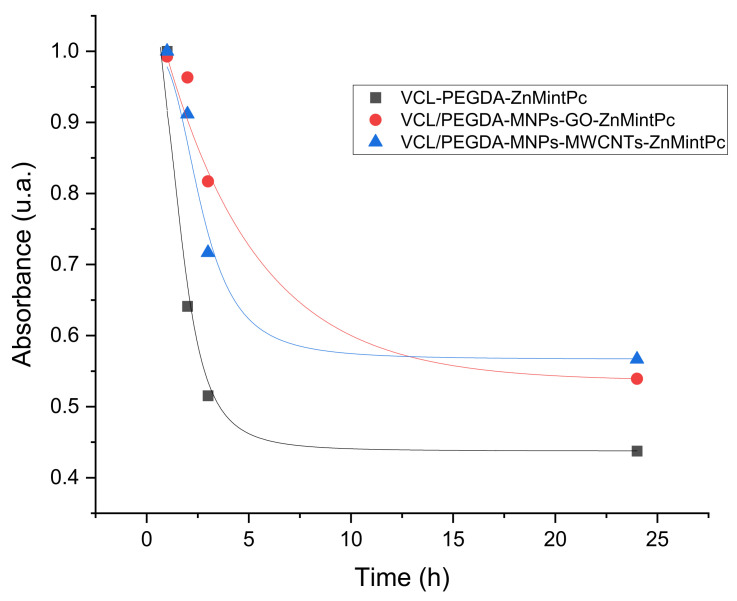
Decay curves of: VCL-PEGDA-ZnMintPc, VCL/PEGDA-MNPs-MWCNTs-ZnMintPc and VCL/PEGDA-MNPs-GO-ZnMintPc. ZnMintPc = 0.27 µM.

The curves showing stability of the magnetics nanocomposites vs. time (Figure 5) indicates that the intensity of PS decays exponentially. The nanocomposites for 24 h were evaluated, and the nanocomposites based on GO and MWCNTs decreased the photobleaching rate of the nanocomposite. Table 2 shows the percentage decay of PS over time that is related to PS release. After 24 h, nanocomposites based on GO and MWCNT only allowed PS decays of 45.67% and 43.33% while, in the hydrogel, the PS decayed 56.24%. Photobleaching in VCL/PEGDA-MNPs-GO-ZnMintPc and VCL/PEGDA-MNPs-MWCNTs-ZnMintPc nanocomposites can be considered very successful.

### 3.4. Photodynamic Analyses

DPBF photobleaching was performed in VCL/PEGDA-MNPs-GO, VCL/PEGDA-MNPs-MWCNTs, VCL/PEGDA-MNPs-GO-ZnMintPc, and VCL/PEGDA-MNPs-MWCNTs-ZnMintPc, to evaluate the efficiency of singlet oxygen production of the nanocomposites using 630 nm light with an intensity of 65.5 mW·cm^−2^. In the Supporting Information (Figures S4 and S5), the decrease of each nanocomposite’s 418 nm DPBF band as a function of time is shown. For the VCL/PEGDA-MNPs-GO (Figure S4a) and VCL/PEGDA-MNPs-MWCNTs (Figure S4b) nanocomposites, a slight decrease in the 418 nm band at an irradiation time of 29 and 35 min, respectively, was observed. On the other hand, for nanocomposites with the presence of PS (VCL/PEGDA-MNPs-GO-ZnMintPc, Figure S5a and VCL/PEGDA-MNPs-MWCNTs-ZnMintPc Figure S5b), rapid decay of the 418 nm band was observed until all the DPBF present was photobleached in solution. Total photobleaching after 9 and 11 min of irradiation, respectively, was observed.

**Figure 6:**
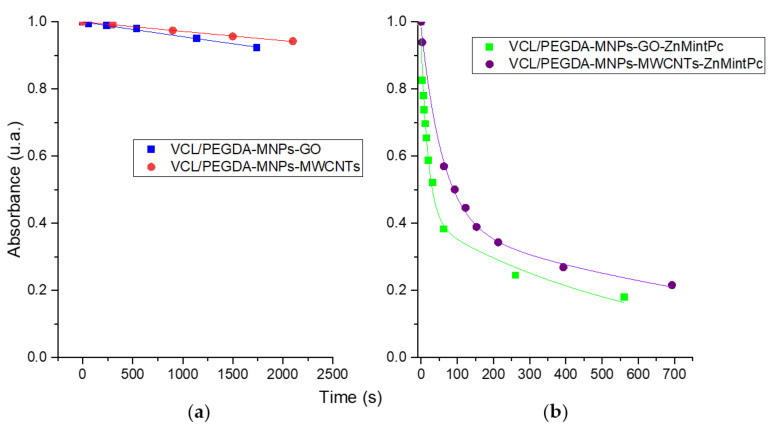
Decay curve of DPBF from (**a**) VCL/PEGDA-MNPs-GO and VCL/PEGDA-MNPs-MWCNTs; (**b**) VCL/PEGDA-MNPs-GO-ZnMintPc and VCL/PEGDA-MNPs-MWCNTs-ZnMintPc. DPBF = 18.5 mM, GO = 3.47 μg·mL^−1^, MWCNTs = 3.47 μg·mL^−1^, MNPs = 93.3 μg·mL^−1^ and ZnMintPc = 8.1 μM.

For each nanocomposite, absorbance vs. photoirradiation curves were constructed. Taking the 418 nm band of DPBF, an exponential decay fit to obtain the decay time presented in Figure 6a,b was made.

Figure 6a shows that the nanocomposites without PS (VCL/PEGDA-MNPs-GO and VCL/PEGDA-MNPs-MWCNTs) have long DPBF photobleaching times. The VCL/PEGDA-MNPs-GO photobleaching time was less than in VCL/PEGDA-MNPs-MWCNTs, indicating that GO has a better capacity to generate ^1^O_2_ by photooxidation of DBPF, as corroborated by the work of Romero et al., 2020 [11]. For nanocomposites with PS (VCL/PEGDA-MNPs-GO-ZnMintPc and VCL/PEGDA-MNPs-MWCNTs-ZnMintPc), curve fitting show photobleaching over two times. The first one indicates a rapid decay in the first 60 s of photoirradiation, and the second one indicates a slow decay from 60 s to 700 s of photoirradiation. Once again, slow decay results in longer photobleaching time, as seen in the purple curve in Table 3 for the MWCNT-based nanocomposite (1140 ± 260 s).

The presence of PS in the nanocomposites generates a rapid photooxidation of DPBF. Therefore, the results indicate that the photobleaching of DPBF was mainly due to PDT effects mediated by VCL/PEGDA-MNPs-GO-ZnMintPc and VCL/PEGDA-MNPs-MWCNTs-ZnMintPc nanocomposites, with the nanocomposite containing GO and the photosensitizing ZnMintPc exhibiting more significant singlet oxygen generation.

### 3.5. Thermal Studies

**Figure 7:**
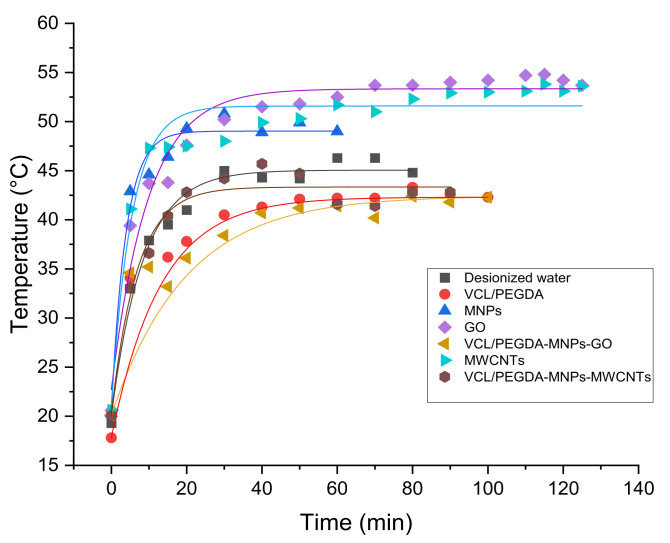
Thermal studies. deionized water (control black line), VCL/PEGDA (red line), MNPs (blue line), GO (violet line), MWCNTs (light blue line), VCL/PEGDA-MNPs-GO (yellow line) and VCL/PEGDA-MNPs-MWCNTs (brown line). GO = 3.47 μg·mL^−1^, MWCNTs = 3.47 μg·mL^−1^, MNPs = 93.3 μg·mL^−1^.

The thermal study curves in Figure 7 show that the solutions of MNPs, GO and MWCNTs (blue, violet and light blue, respectively) act as photothermal materials when irradiated with red light for about 100 min. They can reach temperatures between 50.8 and 54.8 °C, making them suitable for use in Photothermal Therapy due to their ability to convert red and near-infrared (NIR) light into heat, and to transport drugs, as mentioned in the literature [71,117,118,119,120]. In the thermal curves of Figure 7, it can also be observed that VCL/PEGDA, VCL/PEGDA-MNP-GO, and VCL/PEGDA-MNP-MWCNTs nanocomposites irradiated with red light for about 100 min show a slight decrease in temperature compared to the thermal curve of deionized water (reference), and around 80 min of irradiation, curves representing the presence of hydrogel reach temperatures close to deionized water (the light doses are shown in the Table 4).

MNPs, GO, and MWCNTs solutions raised their temperature around 10 °C, relative to the control sample (deionized water) and VCL/PEGDA-MNP-GO and VCL/PEGDA-MNP-MWCNTs nanocomposites. It can be concluded that the VCL/PEGDA hydrogel can absorb a large amount of energy without increasing its temperature. This would be expected because its main components, VCL and PEGDA, have good calorific capacities [121,122,123]. Therefore the hydrogel would inherit this property, which explains why the nanocomposites covered by hydrogel maintain a temperature similar to the temperature of the control sample (deionized water) at an irradiation time of more than 100 min with red light. Around 30 to 45 min, all the nanocomposites reach a threshold temperature that did not change when irradiated for a longer time.

### 3.6. Morphological Studies of Magnetic Nanocomposites

The functionalized VCL/PEGDA-MNPs-GO-ZnMintPc and VCL/PEGDA-MNPs-MWCNTs-ZnMintPc nanocomposites were characterized by SEM, TEM, EDS, and XRD, and the results are presented in Figure 8b,e,f and Figures S6, S9, S11 and S13 for GO-based nanocomposites, in Figure 8a,d and Figures S7, S10, S12 and S14 for MWCNTs-based nanocomposites and in Figure 8c and Figure S8 for free-standing MNPs.

**Figure 8:**
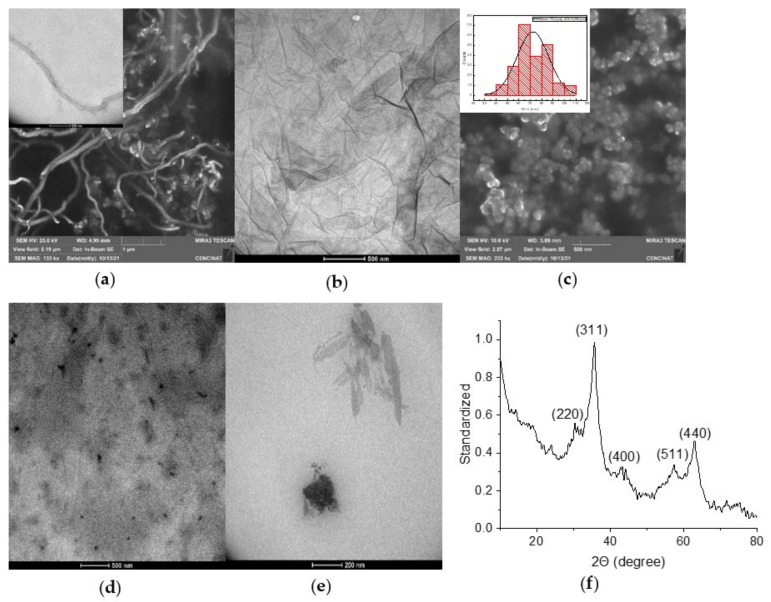
(**a**) SEM and TEM images of purified MWCNTs; (**b**) TEM of GO; (**c**) SEM of MNPs. (**d**) TEM of VCL/PEGDA-MNPs-MWCNTs-ZnMintPc. (**e**) TEM of VCL/PEGDA-MNPs-GO-ZnMintPc; (**f**) XRD analysis of MNPs-GO.

The SEM and TEM images in Figure 8 show the morphology of (a) carbon nanotubes, that have the shape of fibers and in TEM their internal structure and walls, (b) the structure of large sheets of GO. Figure S6d presents the height profile of GO and indicates that the thickness of the GO is roughly 2.8 nm. According to Sun et al., 2010 study [124], this indicates that the sheet is four-layered. Various GO sheet sizes are depicted, but the most common is roughly 2 µm. In Figure 8c, we can observe the MNPs with a spherical shape with an average size of ~72 nm. Figure 8d,e shows the morphology of the hydrogel coating MNPs-GO-ZnMintPc and MNPs-MWCNTs-ZnMintPc, so it is difficult to differentiate the structures covered by the hydrogel. In the Figure 8f, we present the XRD pattern for the MNPs-GO sample, where the diffraction peaks of the iron nanoparticles decorating the GO are observed at 2θ = 30.27°, 35.6°, 43.3°, 53.7°, 57.1° and 63.0°, indicating that the MNPs retain their original crystalline structure after functionalization, agreeing with the results of Amiri, Baghayeri, and Sedighi 2018; Cao et al., 2016 [125,126].

### 3.7. Morphological Studies of Bacteria in Magnetic Nanocomposites

**Figure 9:**
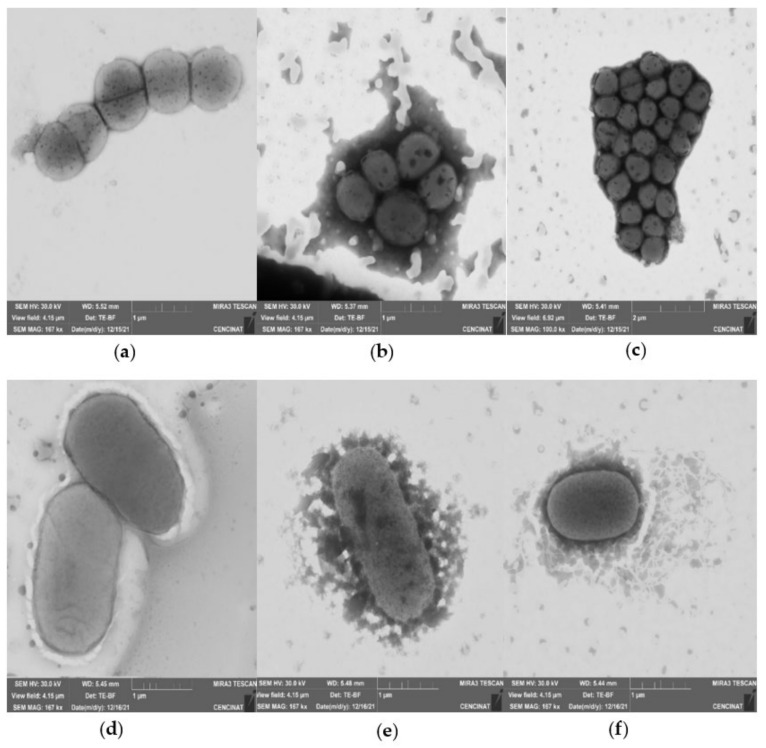
STEM of (**a**) *S. aureus*; (**b**) *S. aureus* + C1 (VCL/PEGDA-MNPs-GO-ZnMintPc); (**c**) *S. aureus* + C2(VCL/PEGDA-MNPs-MWCNTs-ZnMintPc); (**d**) *E. coli*; (**e**) *E. coli* + C1; (**f**) *E. coli* + C2.

To understand the interaction of magnetic nanocomposites with microorganisms, STEM images of *S. aureus* and *E. coli* bacteria were obtained in the presence of VCL/PEGDA-MNPs-GO-ZnMintPc and VCL/PEGDA-MNPs-MWCNT-ZnMintPc nanocomposites (Figure 9b,c,e,f). In the STEM image of *S. aureus* and *E. coli* pure (Figure 9a,d), their structure and morphology are not altered, and the membrane covers them without ruptures. When interacting with the VCL/PEGDA-MNPs-GO-ZnMintPc and VCL/PEGDA-MNPs-MWCNT-ZnMintPc nanocomposites, no changes were observed in the morphology of the bacteria (images taken 24 h after the bacterial-nanocomposite solutions were prepared) [127,128,129]. It was possible to observe how the nanocomposite can completely cover the microorganism, allowing the nanocomposites dispersed in the hydrogel to be photoexcited with the red-light source and cause microbial elimination.

### 3.8. Antimicrobial Effect of Magnetic Nanocomposites

The antimicrobial effect of the VCL/PEGDA-MNPs-GO-ZnMintPc (C1), VCL/PEGDA-MNPs-MWCNTs-ZnMintPc (C2), MNPs-GO (C3), and MNPs-MWCNTs (C4) nanocomposites were evaluated while irradiating with 630 nm red light at 65 mW cm^−2^ in the presence of the microorganisms *S. aureus*, *E. coli* and *C. albicans*.

**Figure 10:**
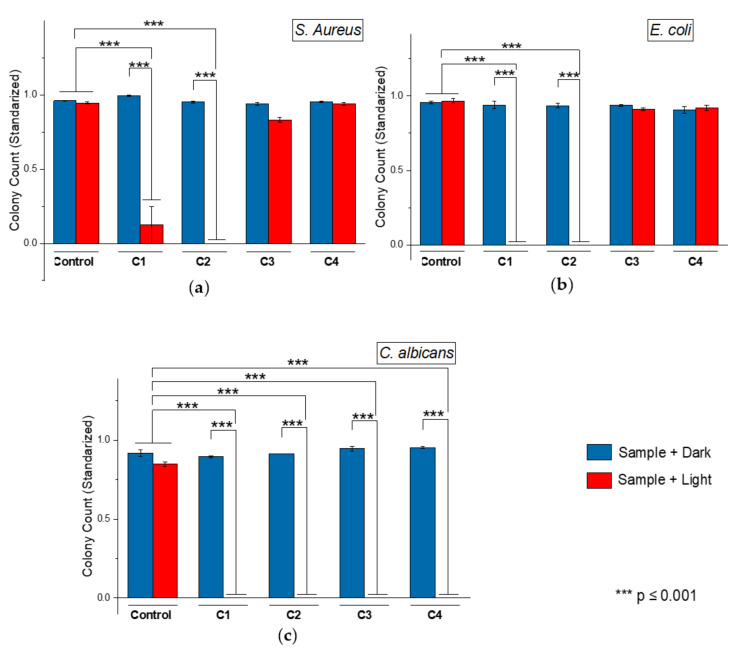
PDT/PTT antimicrobial effect. Standardized result of LOG(UFC/mL) by (**a**) *S. aureus;* (**b**) *E. coli* and (**c**) *C. albicans* based in C1, C2, C3 and C4 nanocomposites. The concentration of MNPs in C1, C2, C3 and C4 was 93.3 μg·mL^−1^. The concentration of ZnMintPc in C1 and C2 was 8.1 μM. The concentration of GO in C1 and C3 was 3.47 μg·mL^−1^. The concentration of MWCNTs in C2 and C4 was 3.47 μg·mL^−1^. Time of irradiation of C1 and C2 nanocomposites was 30 min and for C3 and C4 was 40 min. Significant differences in means according to the Tukey test (*** *p* ≤ 0.001).

**Figure 11:**
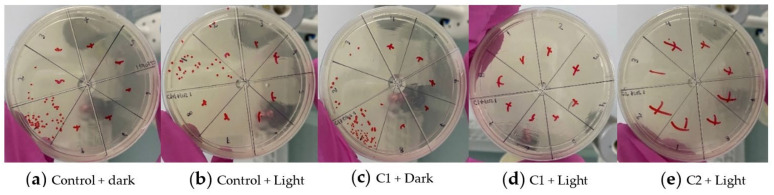
Antimicrobial effect of C1 and C2 nanocomposites at *C. albicans* in vitro. C1: GO-MNPs-VGLPEGDA- ZnMintPc, C2: MWCNT-MNPs-VGLPEGDA- ZnMintPc, C3: GO-MNPs-VGLPEGDA, C4: MWCNT-MNPs-VGLPEGDA. The concentration of MNPs in C1, C2, C3 and C4 was 93.3 μg·mL^−1^. The concentration of ZnMintPc in C1 and C2 was 8.1 μM. The concentration of GO in C1 and C3 was 3.47 μg·mL^−1^. The concentration of MWCNTs in C2 and C4 was 3.47 μg·mL^−1^.

The results obtained for each colony are shown in Figure 10. The histograms present the LOG (CFU mL^−1^) evaluation standardized to 1. A concentration of 10^7^ CFU mL^−1^ for *S. aureus*, 10^6^ CFU mL^−1^ for *E. coli*, and 10^5^ CFU mL^−1^ for *C. albicans* was used. Results show that light alone cannot eliminate microorganisms (control+light samples), as in Figure 11 with *C. albicans*. To eliminate the microorganism, it was necessary to irradiate it in the presence of a nanocomposite (C1 + light or C2 + light).

The quantitative result of colony counts of *S. aureus*, *E. coli*, and *C. albicans* after being irradiated with a red LED (630 nm 65 mW cm^−2^) indicates that for the colonies of *S. aureus* and *E. coli* (Figure 10a,b), samples C3, C4 in light and dark, were not different from the control group. In contrast, the count in samples C1 + light and C2 + light was significantly lower than the control group (*** *p* ≤ 0.001). The colony count of *C. albicans* in the samples + dark was not different from the control group, but the count in the samples C1 + light, C2 + light, C3 + light, and C4 + light was significantly lower than the control group (*** *p* ≤ 0.001). This means that after irradiation, the C1 nanocomposite eliminated all *E. coli*, and *C. albicans*, and some *S. aureus*. C2 nanocomposite eliminated *S. aureus*, *E. coli*, and *C. albicans*. C3 and C4 nanocomposites eliminated all *C. albicans*. Therefore, all nanocomposites can eliminate some of the microorganisms used in this study, with C2 being the best due to its ability to eliminate the three types of microorganisms. This agrees with the results obtained by Huo et al., 2021; Liu et al., 2021 and Ren et al., 2020 [129,130,131].

In Figure 10c, the samples without hydrogel C3 + light and C4 + light only had a complete response with *C. albicans*, and it could be considered that the photothermic effect allowed microbial elimination. This is in agreement with dos Santos et al., 2019 [132]. This conclusion is based on the results shown in Figure 7 of the photothermal effect for compounds based on MWCNT, MNP, GO, where it is observed that their temperature increased as the irradiation time increased. For the samples with hydrogel and photosensitizer (C1 + light and C2 + light), it can be concluded that the elimination of the microorganisms *S. aureus* (Figure 10a), *E. coli* (Figure 10b), and *C. albicans* (Figure 10c) was produced by the photodynamic effect, as supported by the research of Mei et al., 2021 [12] as well as the data reported by Xu, Yao and Xu 2019 [52]. This conclusion is based on the results in Table 3 that show that compounds containing ZnMintPc have a longer decay period than those which do not.

According to the results obtained, the VCL/PEGDA-MNPs-GO-ZnMintPc and VCL/PEGDA-MNPs-MWCNTs-ZnMintPc nanocomposites have a ferromagnetic character, typical of nanocomposites with iron nanoparticles and with low saturation magnetization, due to being covered by a diamagnetic hydrogel layer, in agreement with the studies carried out by Donadel et al., 2008, Mahdavi et al., 2013, and Qu et al., 2010 [133,134,135]; in which MNPs were synthesized for bioapplications and it was demonstrated that the surface modification caused a reduction in saturation magnetization, with values between 67 to 22 emu·g^−1^ depending on the type of biopolymer to be used.

Due to the physical and magnetic properties of these nanocomposites, it was shown that they could avoid the early extinction of the fluorescence of PS ZnMintPc, thus improving their photodynamic effect, as mentioned in the work of Huang et al., 2011 and Xiao et al., 2021. The thermodynamic studies carried out by Kou et al., 2019, Srivastava and Kumar, 2010, and Tager et al., 1993 indicate that VCL and PEGDA have good heat capacities of around 258 and 94 J·mol^−1^·K^−1^, which is why they are usually used to synthesize cryogels. This explains that the nanocomposites covered by the VCL/PEGDA hydrogel are capable of being maintained up to a maximum temperature of 40 °C receiving doses of red light of up to 393 J·cm^−2^, which in comparison to nanocomposites without hydrogel, raise their temperature to around 55 °C, as described in the literature [71,117,118,119,120].

The internalization mechanism of these nanocomposites occurs through direct contact between the microorganism-nanocomposite and the PTT/PDT effect, as seen in Figure 9. Several authors have discussed the mechanism of programmed cell death due to the PDT/PTT effect, and as mentioned by Buzzá et al., 2021, Patil et al., 2021, among others in their scientific articles, the ability of PS, GO, and MWCNT to locate in various organelles and the action of the PTT/PDT effect promotes ROS generation followed by physical damage to the membrane. Oxidative stress leads to changes in calcium and lipid metabolism, generating cytokines and stress response mediators that lead to induction of apoptosis by the mitochondrial pathway and specific protein oxidation [136,137,138,139,140]. It can be concluded that this mechanism is the cause of the death of the microorganisms *S. aureus*, *E. coli*, and *C. albicans* in the present study. The results shown in Figure 10c indicate the elimination of *C. albicans* by the PTT effect and, as mentioned by Mocan et al., 2014, and Pérez-Hernández et al., 2015 in their research, PTT causes apoptosis, or programmed cell death, rather than necrotic cell death, by activating the intrinsic route. Inflammatory reactions are triggered by necrotic cell death, carbon-based nanomaterials in the photothermal treatment activate the flux of free radicals within the cell, and the oxidative state mediates cellular damage in PC cells via apoptotic pathway [141,142]. Therefore, it can be concluded that this is the mechanism that causes the death of *C. albicans* when there is no presence of PS.

The excretion pathway of these nanocomposites in living systems has been investigated by authors such as Dias et al., 2021 and Liu et al., 2008, who indicate that these nanocomposites based on carbon materials and MNPs can be excreted through the biliary and urinary tracts [143,144].

## 4. Conclusions

The present study involved synthesis of magnetic nanocomposites VCL/PEGDA-MNPs-MWCNTs-ZnMintPc and VCL/PEGDA-MNPs-GO-ZnMintPc to eliminate three types of microbial colonies: *S. aureus*, *E. coli* and *C. albicans*.

After these nanocomposites were synthesized, optical, magnetic, and morphological characterizations showed that GO, MWCNTS, iron MNPS and ZnMintPc are covered by VCL/PEGDA hydrogel.

The optical properties of these nanocomposites allow them to prevent the rapid disintegration of PS, which is essential in PDT.

Using photodynamic analysis, the nanocomposites the applicability in PDT of VCL/PEGDA-MNPs-MWCNTs-ZnMintPc and VCL/PEGDA-MNPs-GO-ZnMintPc were with low dose red light. It was observed that the nanocomposite VCL/PEGDA-MNPs-GO- ZnMintPc had higher efficiency than the nanocomposite based on MWCNTs since it produced faster photobleaching of DPBF because it is capable of transporting a more significant amount of ZnMintPc and MNPs due to its large specific area. In addition, GO contributed to PS in the formation of ^1^O_2_. Since the nanocomposites are coated with a hydrogel, they are also suitable for controlled PS release systems, with promising applications for PDT.

The nanocomposite that contains GO and PS ZnMintPc had higher efficiency since it produced faster photobleaching of DPBF because it is capable of transporting a more significant amount of ZnMintPc and MNPs due to its large specific area. In addition, the GO contributed to PS in the formation of ^1^O_2_.

Finally, we demonstrated that the VCL/PEGDA-MNPs-GO-ZnMintPc nanocomposite was able to eliminate colonies of *E. coli* and *C. albicans*, and the VCL/PEGDA-MNPs-MWCNTs-ZnMintPc nanocomposite eliminated the three types of microorganisms, which can therefore be considered as a broad-spectrum antimicrobial agent in PDT and PTT.

## Figures and Tables

**Table 1:** Magnetic parameters for the three studied samples: saturation magnetization at *T*_0_ = −273.15 °C (*M*_0_), saturation magnetization *M_S_* at 20 °C, a fraction of the volume of the nanoparticles that remain ferromagnetic (f), and spin-wave constant (B).

Sample	*M*_0_ (emu·g^−1^)	*M_S_* @ 20 °C (emu·g^−1^)	*f* (%)	*B* (°C^−3/2^)
MNPs	52.1	40.1	23.5	4.5 × 10^−5^
VCL-PEGDA-MNPs-MWCNTs	25.5	17.9	11.5	5.8 × 10^−5^
VCL-PEGDA-MNPs-GO	15.9	9.3	7.2	8.3 × 10^−5^

**Table 2:** Decay of ZnMintPc in nanocomposites in 24 h.

Time (h)	VCL-PEGDA-ZnMintPc		VCL/PEGDA-MNPs-GO-ZnMintPc		VCL/PEGDA-MNPs-MWCNTs-ZnMintPc	
	Absorbance	PS Released	Absorbance	PS Released	Absorbance	PS Released
0	100%	-	100%	-	100%	-
1	64.13%	35.87%	97.02%	2.98%	91.17%	8.83%
2	51.55%	48.55%	82.30%	17.7%	71.68%	28.32%
24	43.76%	56.24%	54.33%	45.67%	56.67%	43.33%

**Table 3:** Decay times of nanocomposites.

**VCL/PEGDA-MNPs-MWCNTs**	**VCL/PEGDA-MNPs-MWCNTs-ZnMintPc**
34700 ± 700 s	1140 ± 260 s
**VCL/PEGDA-MNPs-GO**	**VCL/PEGDA-MNPs-GO-ZnMintPc**
22300 ± 900 s	630 ± 190 s

**Table 4:** Light dose applied in the nanocomposites.

Time (min)	Light Dose (J·cm^−2^)
0	0
10	39.3
20	78.6
30	117.9
40	157.2
50	196.5
60	235.8
70	275.1
80	314.4
90	353.7
100	393

## Supplementary Materials

The following supporting information can be downloaded at: https://www.mdpi.com/article/10.3390/pharmaceutics14040705/s1, Figure S1. Optical properties: (a) UV-VIS spectra of DMF(3mL) with different concentrations of ZnMintPc, (b) UV-VIS spectra of VCL/PEGDA(3mL) with different concentrations of ZnMintPc, (c). UV-VIS spectra of VCL/PEGDA (3mL) with MNPs-MWCNTs at different concentrations. (d). VCL/PEGDA with MNPs-MWCNTs and ZnMintPc at different concentrations; Figure S2. Optical properties of Magnetic Nanocomposites: (a) UV-VIS spectra of VCL/PEGDA (3mL) with different concentrations of MNPs-GO, (b) UV-VIS spectra of VCL/PEGDA (3mL) with MNPs-GO-ZnMintPc at different concentrations; Figure S3. (a) Composites calibration curve of ZnMintPc (0.52 µM) from: PS in DMF, VCL/PEGDA, VCL/PEGDA-MNPs-GO and VCL/PEGDA-MNPs-MWCNTs; (b) Absorbance of ZnMintPc-DMF and nanocomposites; Figure S4. Photodynamic Analyses of: (a) UV-VIS spectra of VCL/PEGDA-MNPs-GO and (b) UV-VIS spectra of VCL/PEGDA-MNPs-MWCNTs; Figure S5. Photodynamic Analyses of: (a) UV-VIS spectra of VCL/PEGDA-MNPs-GO-ZnMintPc and (b) UV-VIS spectra of VCL/PEGDA-MNPs-MWCNTs-ZnMintPc; Figure S6: (a) SEM analysis, (b) TEM analysis, (c) Atomic force microscopy (AFM) image (d)Height profile along the line of the panel from GO (e) EDS analysis and (f) XRD of GO; Figure S7. (a) SEM, (b) TEM, (c) EDS and (d) XRD analysis of MWCNTs; Figure S8. (a) SEM, (b) TEM, (c) EDS and (d) XRD analysis of MNPs; Figure S9. (a) SEM, (b) TEM, (c) EDS and (d) XRD analysis of MNPs-GO; Figure S10. (a) SEM, (b) TEM, (c) EDS and (d) XRD analysis of MNPs-MWCNTs; Figure S11: (a) SEM, (b) TEM, (c) EDS analysis of VCL/PEGDA-MNPs-GO; Figure S12: (a) SEM, (b) TEM, (c) EDS analysis of VCL/PEGDA-MNPs-MWCNTs; Figure S13. (a) SEM, (b) TEM, (c) EDS and (d) XRD analysis of VCL/PEGDA-MNPs-GO-ZnMintPc; Figure S14. (a) SEM, (b) TEM, (c) EDS and (d) XRD analysis of VCL/PEGDA-MNPs-MWCNTs-ZnMintPc.
