# Supplementary material for: Broad-Spectrum Antimicrobial ZnMintPc Encapsulated in Magnetic-Nanocomposites with Graphene Oxide/MWCNTs Based on Bimodal Action of Photodynamic and Photothermal Effects
Source: Pharmaceutics. 2022 Mar 26;14(4):705. doi: 10.3390/pharmaceutics14040705 (PMC9028436; doi:10.3390/pharmaceutics14040705)
Supplement: Supplementary file 1 [file pharmaceutics-14-00705-s001.zip › pharmaceutics-1561011-supplementary.pdf]

pharmaceutics

Supplementary Material

Broad-Spectrum Antimicrobial ZnMintPc Encapsulated in Magnetic-Nanocomposites with Graphene Oxide/MWCNTs Based on Bimodal Action of Photodynamic and Photothermal Effects

Coralia Fabiola Cuadrado, Antonio Díaz-Barrios, Kleber Orlando Campaña, Eric Cardona Romani, Francisco Quiroz, Stefania Nardecchia, Alexis Debut, Karla Vizuete, Dario Niebieskikwiat, Camilo Ernesto Ávila, Mateo Alejandro Salazar, Cristina Garzón-Romero, Ailín Blasco-Zúñiga, Miryan Rosita Rivera and María Paulina Romero

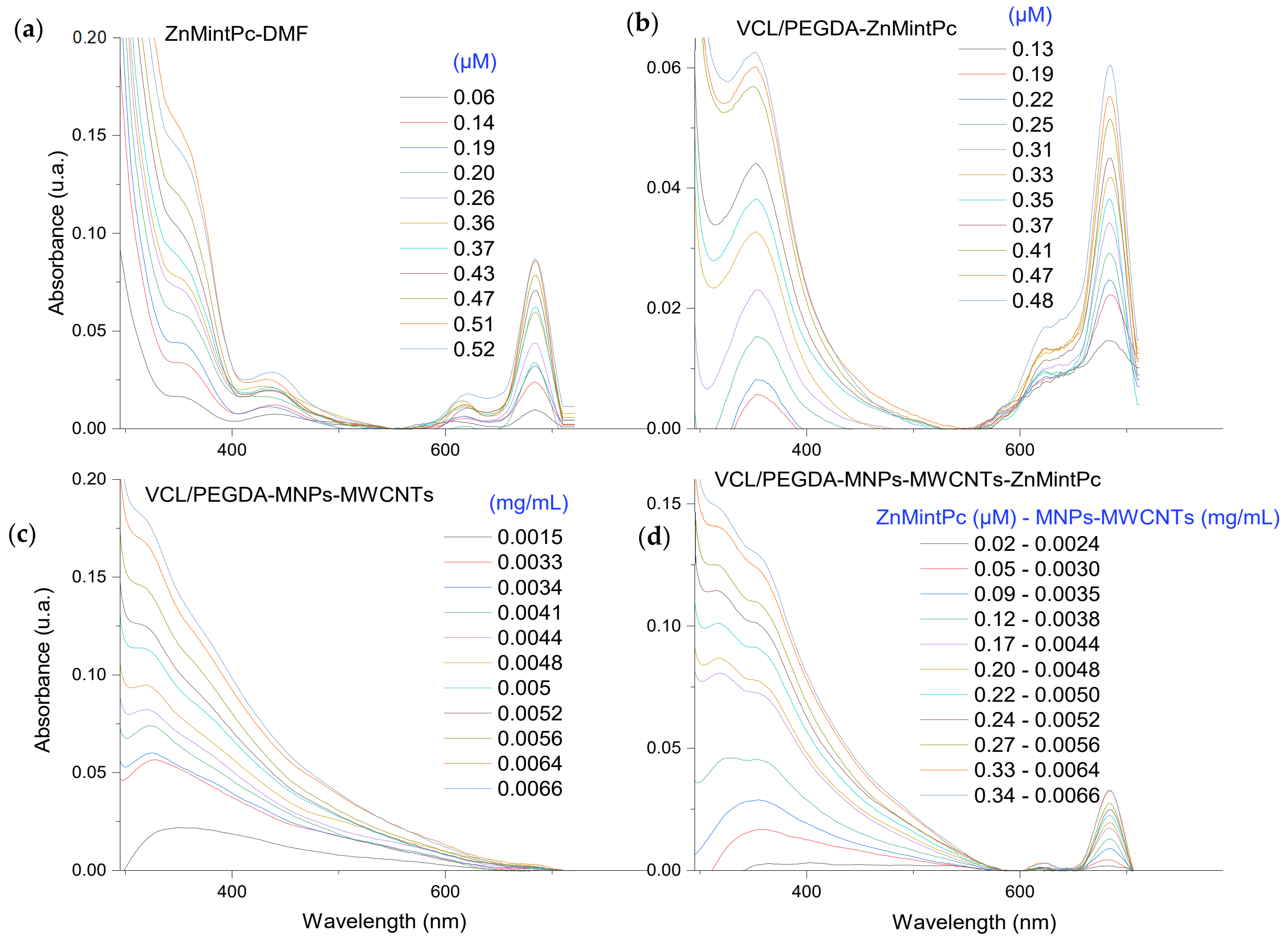

Figure S1. Optical properties: (**a**) UV-VIS spectra of DMF(3mL) with different concentrations of ZnMintPc, (**b**) UV-VIS spectra of VCL/PEGDA(3mL) with different concentrations of ZnMintPc, (**c**). UV-VIS spectra of VCL/PEGDA (3mL) with MNPs-MWCNTs at different concentrations. (**d**). VCL/PEGDA with MNPs-MWCNTs and ZnMintPc at different concentrations.

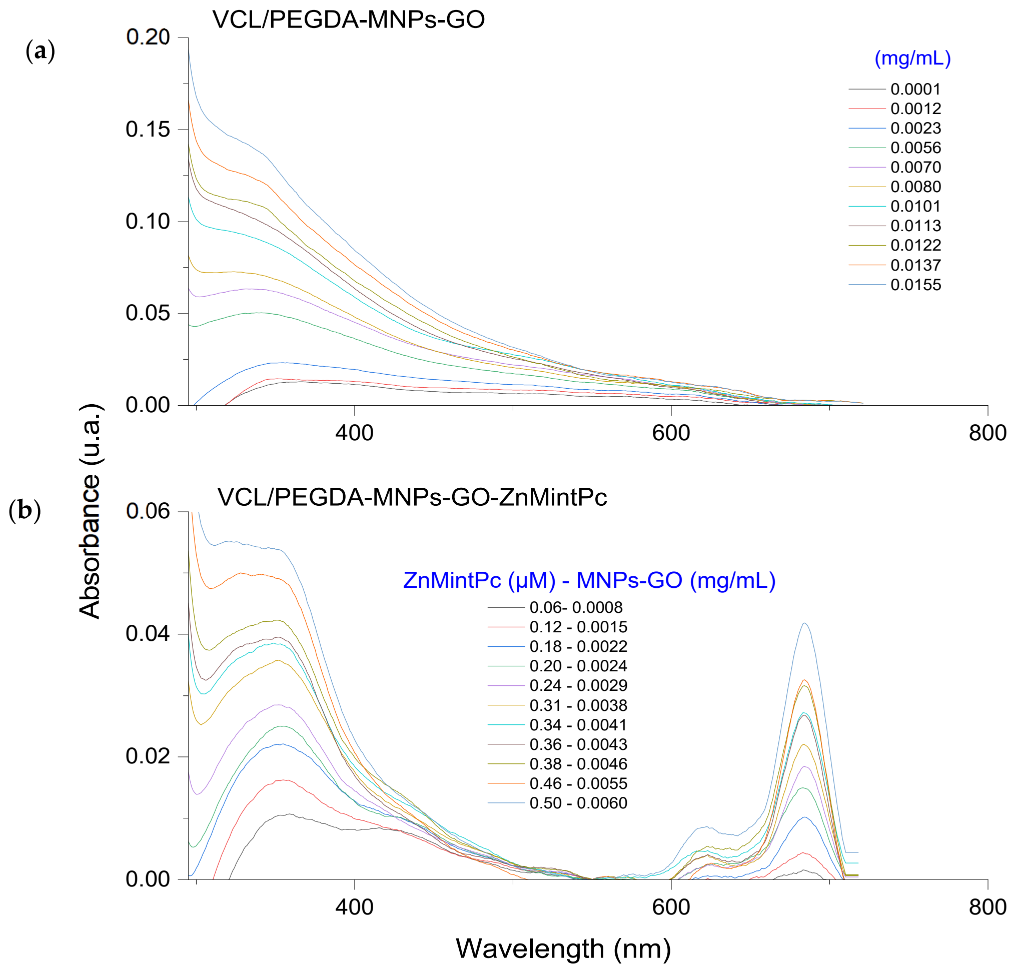

Figure S2. Optical properties of Magnetic Nanocomposites: (**a**) UV-VIS spectra of VCL/PEGDA (3mL) with different concentrations of MNPs-GO, (**b**) UV-VIS spectra of VCL/PEGDA (3mL) with MNPs-GO-ZnMintPc at different concentrations.

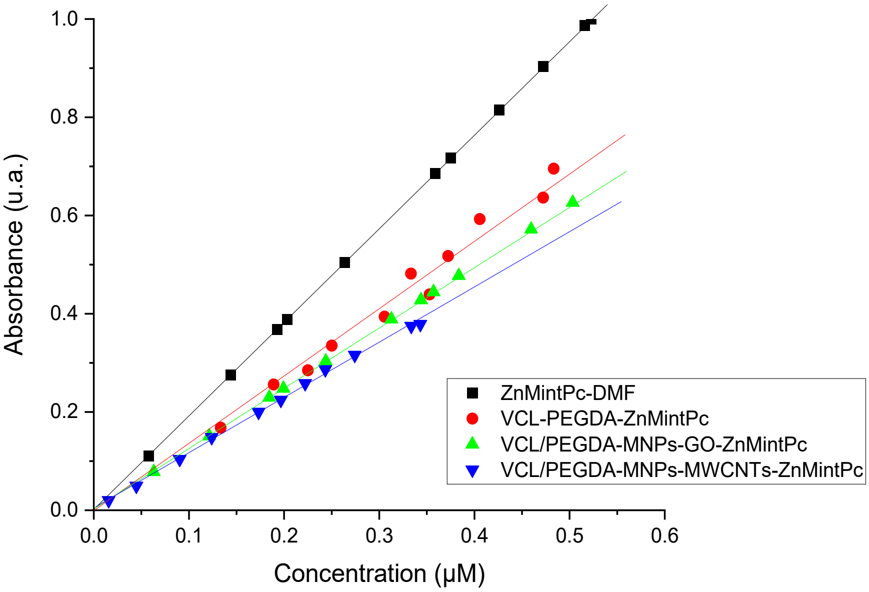

(**a**)

	**Absorbance**
**ZnMintPc-DMF**	100%
**VCL/PEGDA-ZnMintPc**	71.47%
**VCL/PEGDA-MNPs-GO-ZnMintPc**	64.46%
**VCL/PEGDA-MNPs-MWCNTs-ZnMintPc**	61.73%

(**b**)

Figure S3. (**a**) Composites calibration curve of ZnMintPc (0.52 μM) from: PS in DMF, VCL/PEGDA, VCL/PEGDA-MNPs-GO and VCL/PEGDA-MNPs-MWCNTs; (**b**) Absorbance of ZnMintPc-DMF and nanocomposites.

(**a**)

(**b**)

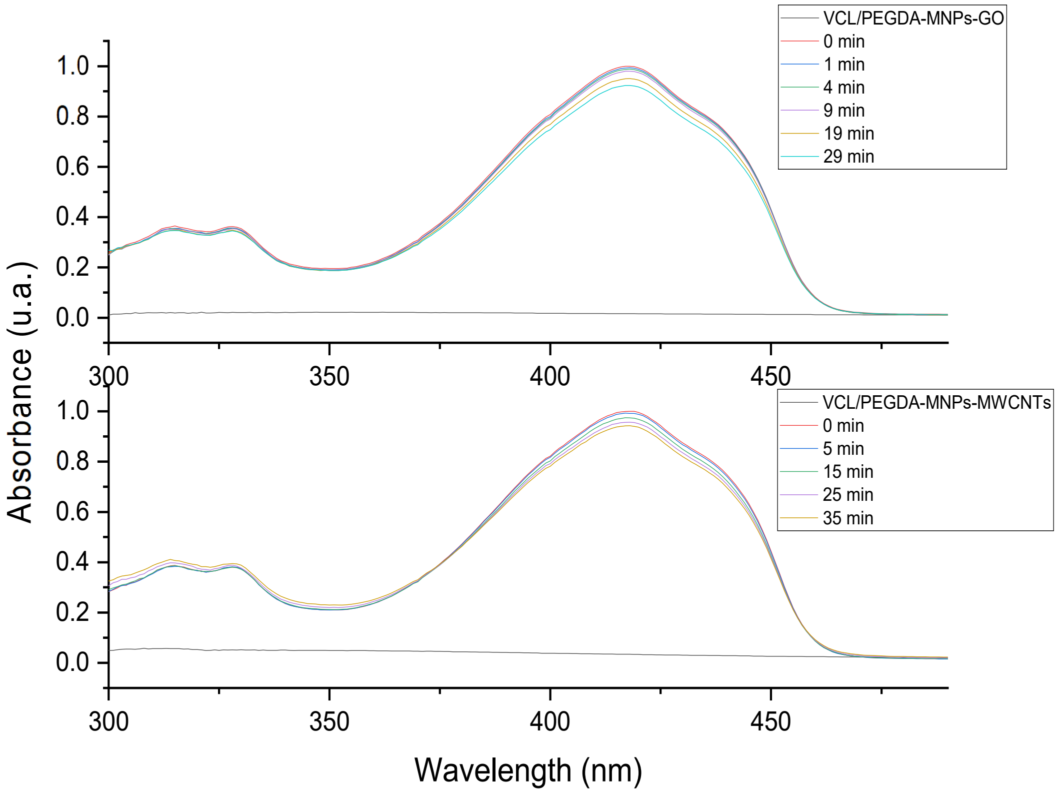

Figure S4. Photodynamic Analyses of: (**a**) UV-VIS spectra of VCL/PEGDA-MNPs-GO and (**b**) UV-VIS spectra of VCL/PEGDA-MNPs-MWCNTs.

(**a**)

(**b**)

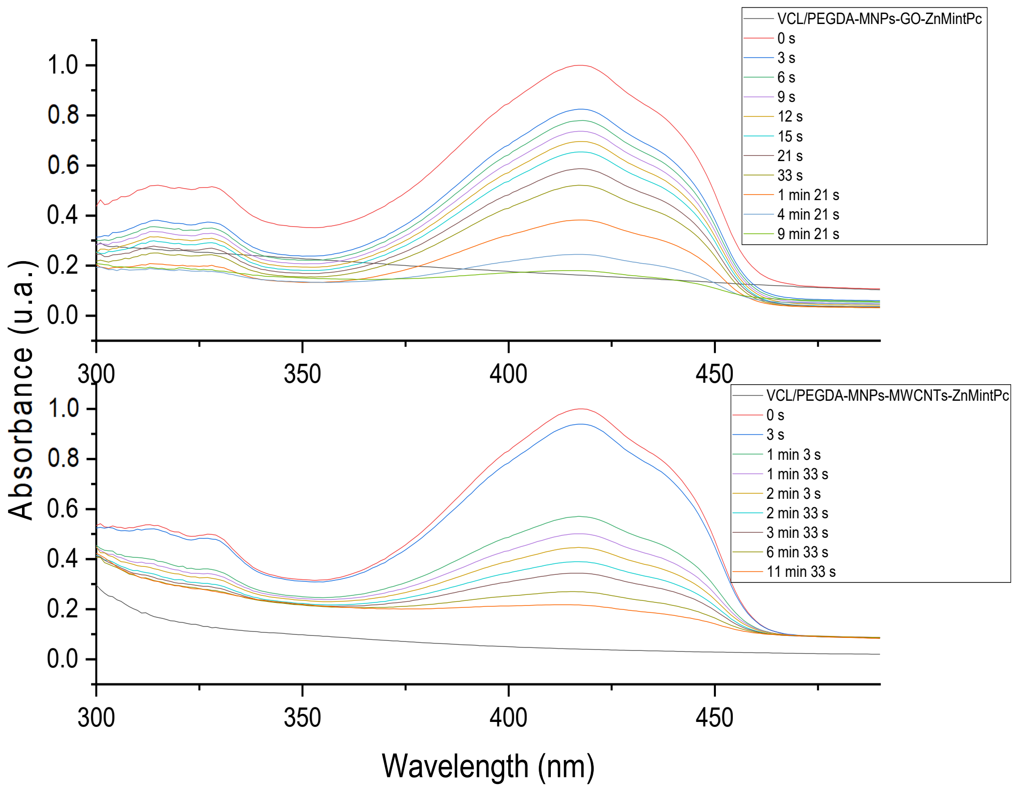

Figure S5. Photodynamic Analyses of: (**a**) UV-VIS spectra of VCL/PEGDA-MNPs-GO-ZnMintPc and (**b**) UV-VIS spectra of VCL/PEGDA-MNPs-MWCNTs-ZnMintPc.

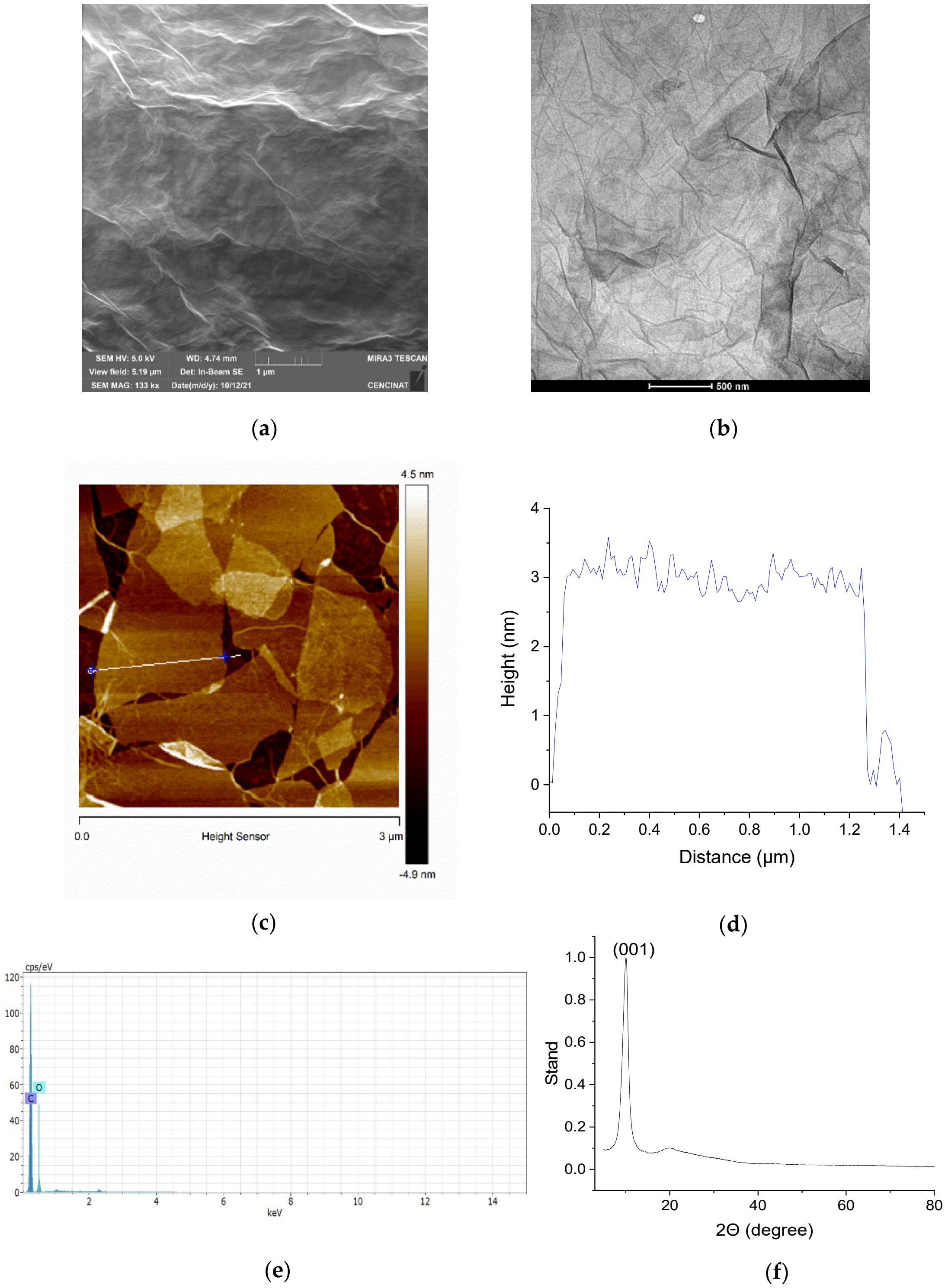

Figure S6. (**a**) SEM analysis, (**b**) TEM analysis, (**c**) Atomic force microscopy (AFM) image (**d**)Height profile along the line of the panel from GO (**e**) EDS analysis and (**f**) XRD of GO.

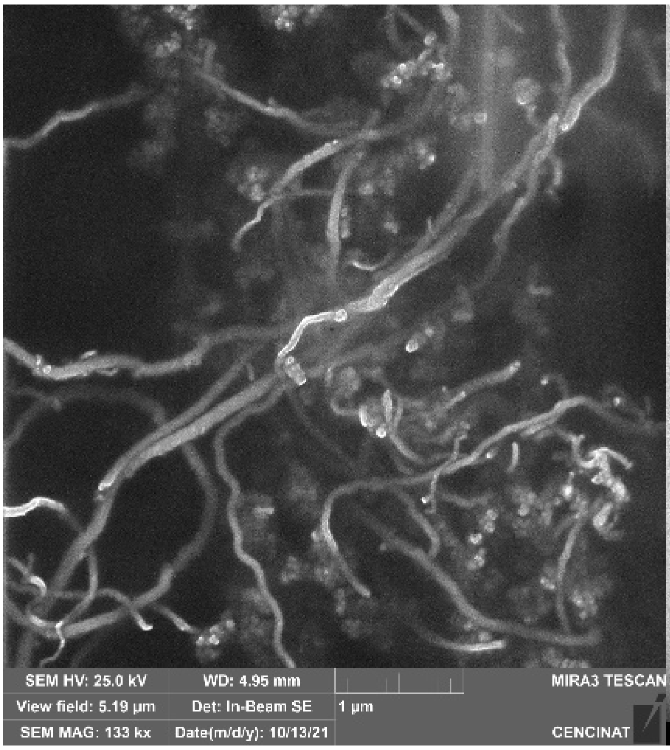

(**a**)

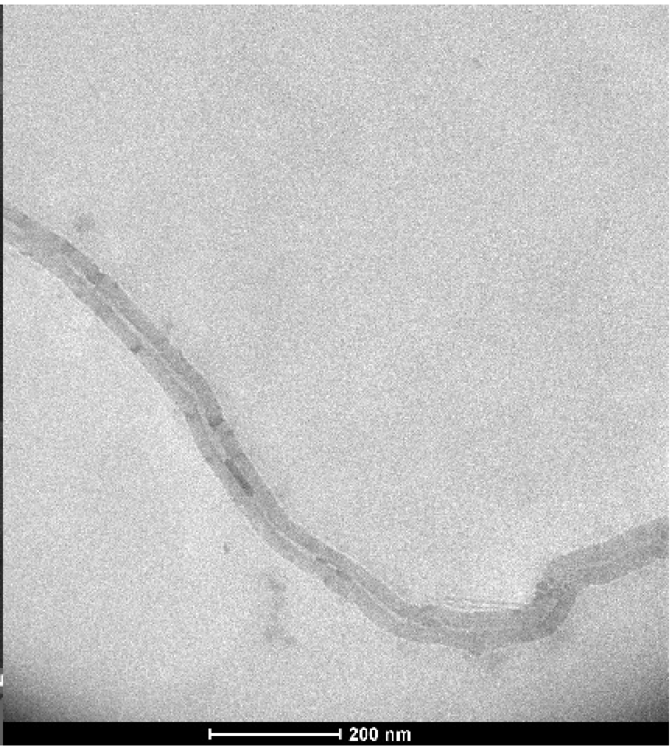

(**b**)

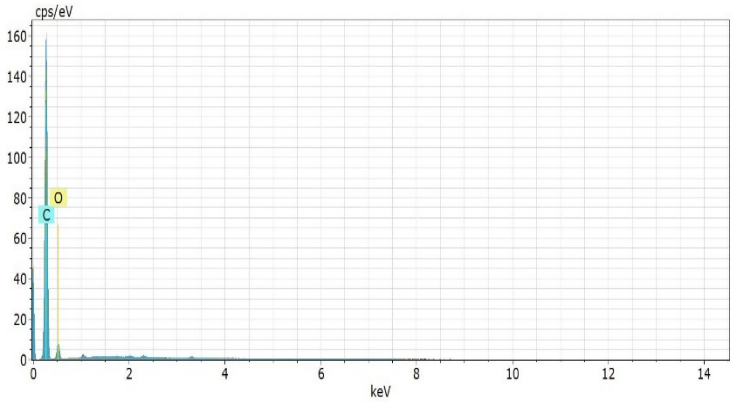

(**c**)

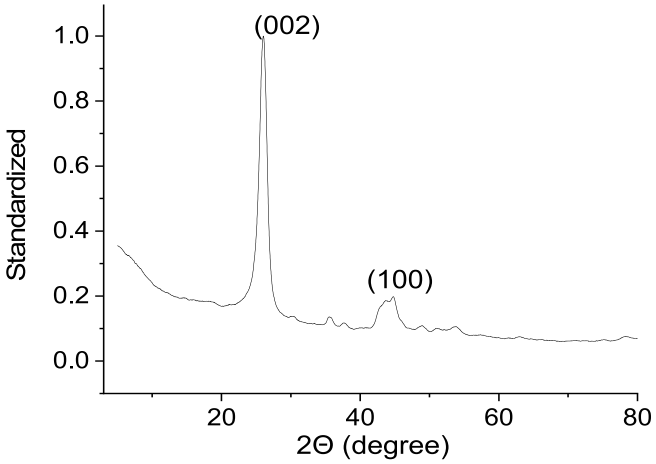

(**d**)

Figure S7. (**a**) SEM, (**b**) TEM, (**c**) EDS and (**d**) XRD analysis of MWCNTs.

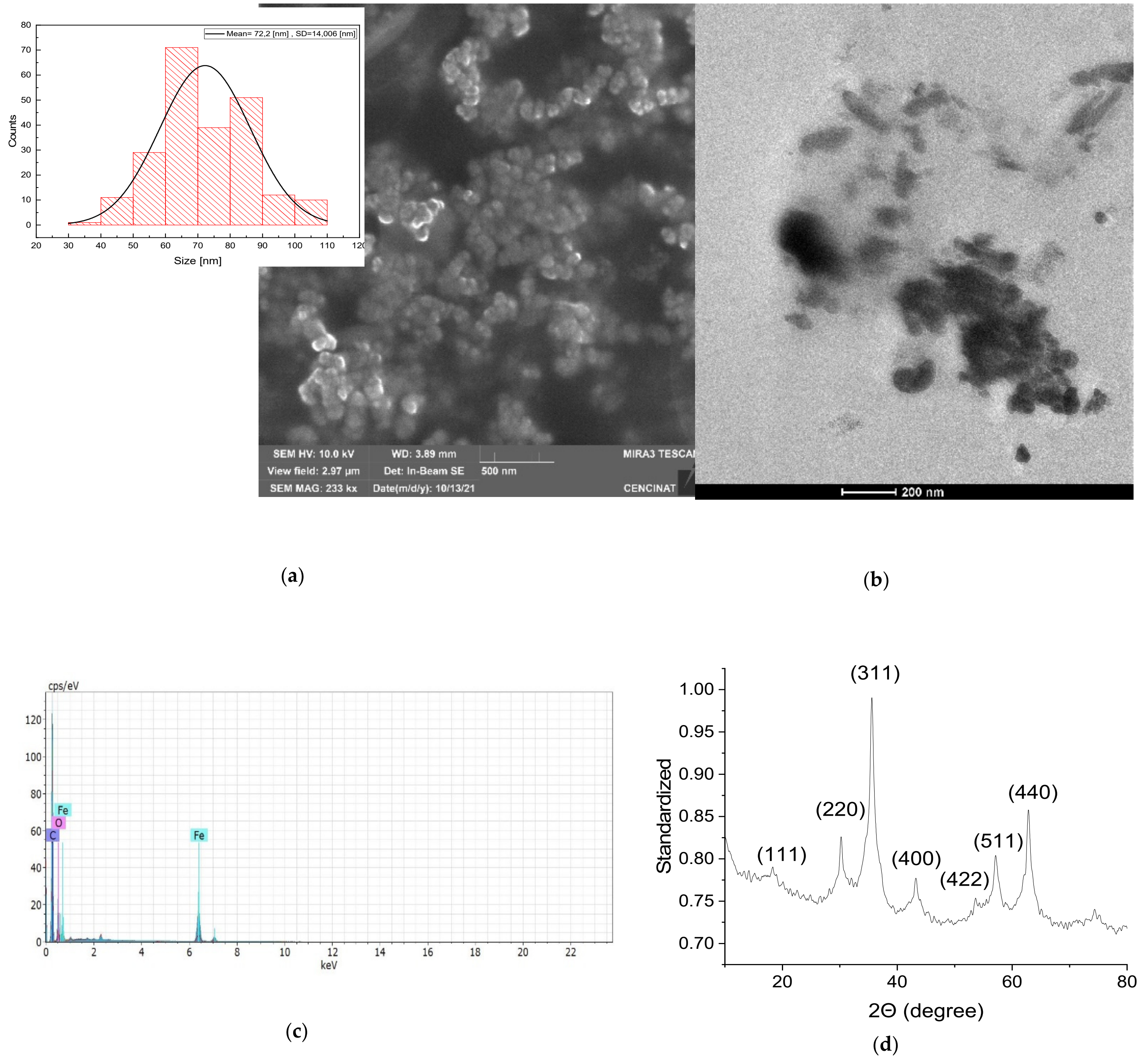

Figure S8. (**a**) SEM, (**b**) TEM, (**c**) EDS and (**d**) XRD analysis of MNPs.

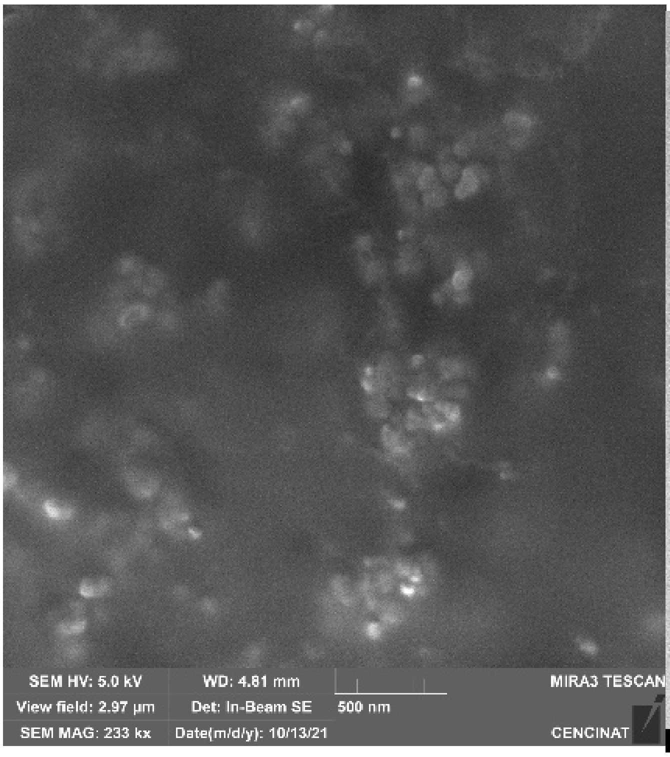

(**a**)

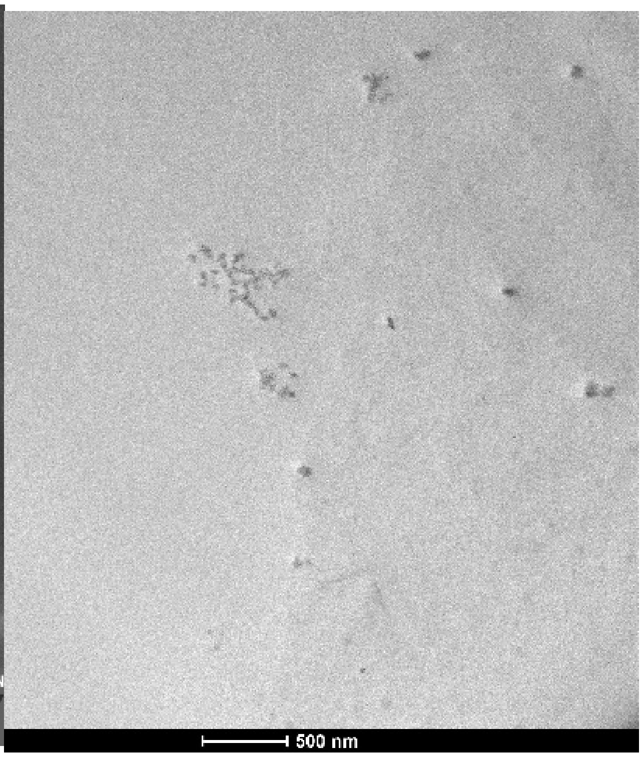

(**b**)

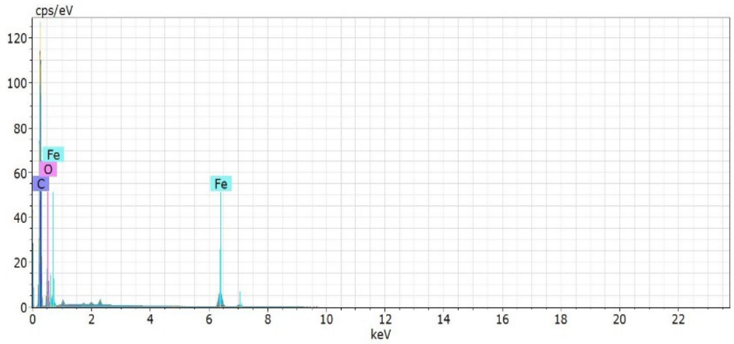

(**c**)

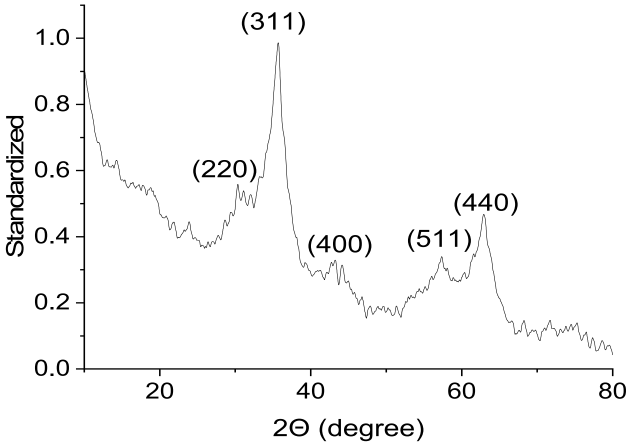

(**d**)

Figure S9. (**a**) SEM, (**b**) TEM, (**c**) EDS and (**d**) XRD analysis of MNPs-GO.

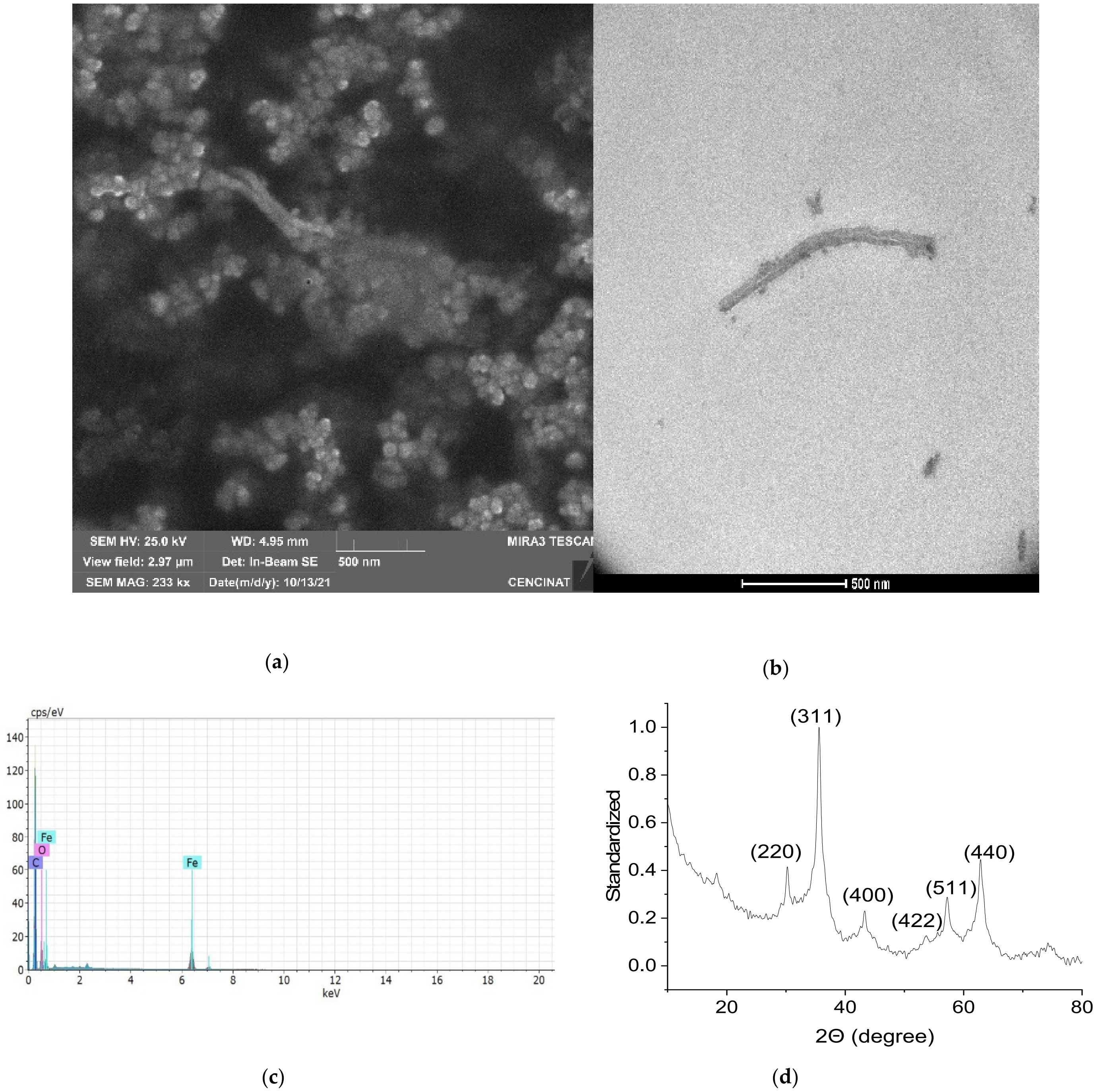

Figure S10. (**a**) SEM, (**b**) TEM, (**c**) EDS and (**d**) XRD analysis of MNPs-MWCNTs.

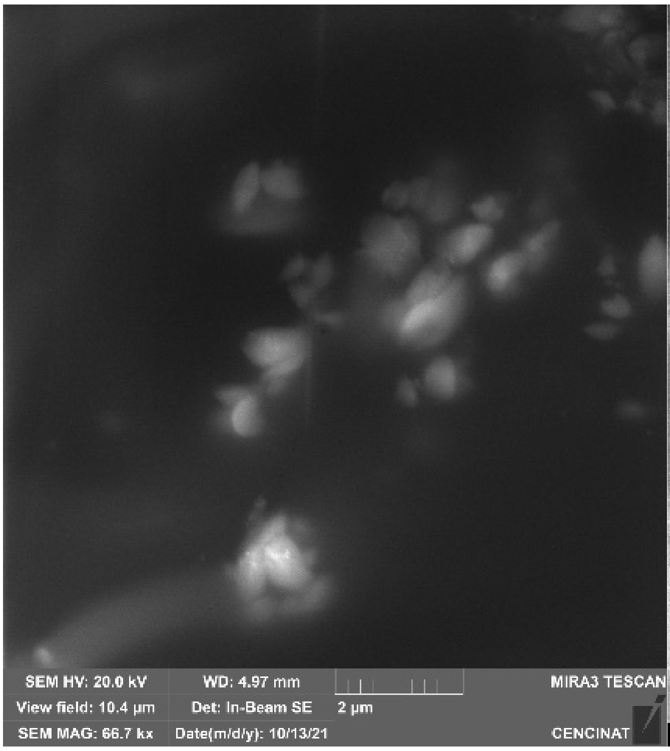

(**a**)

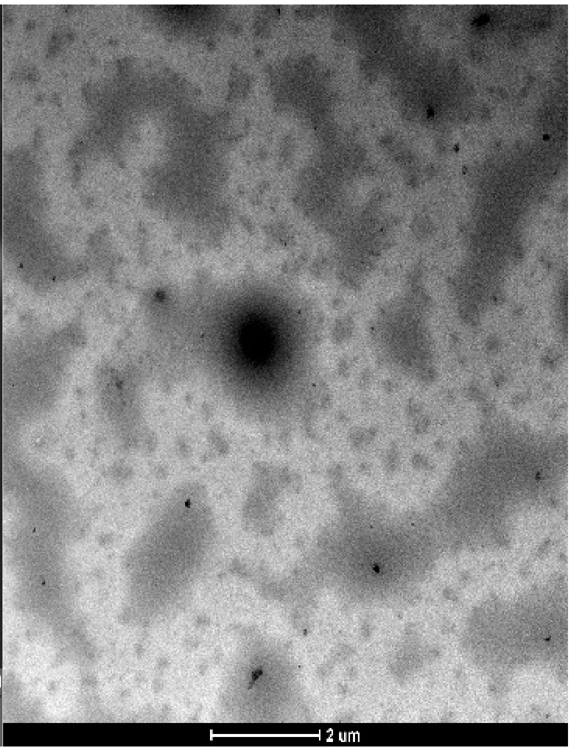

(**b**)

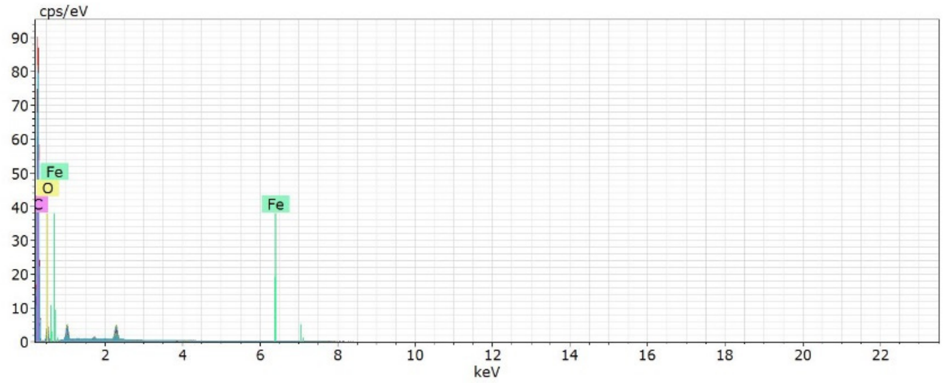

(**c**)

Figure S11. (**a**) SEM, (**b**) TEM, (**c**) EDS analysis of VCL/PEGDA-MNPs-GO.

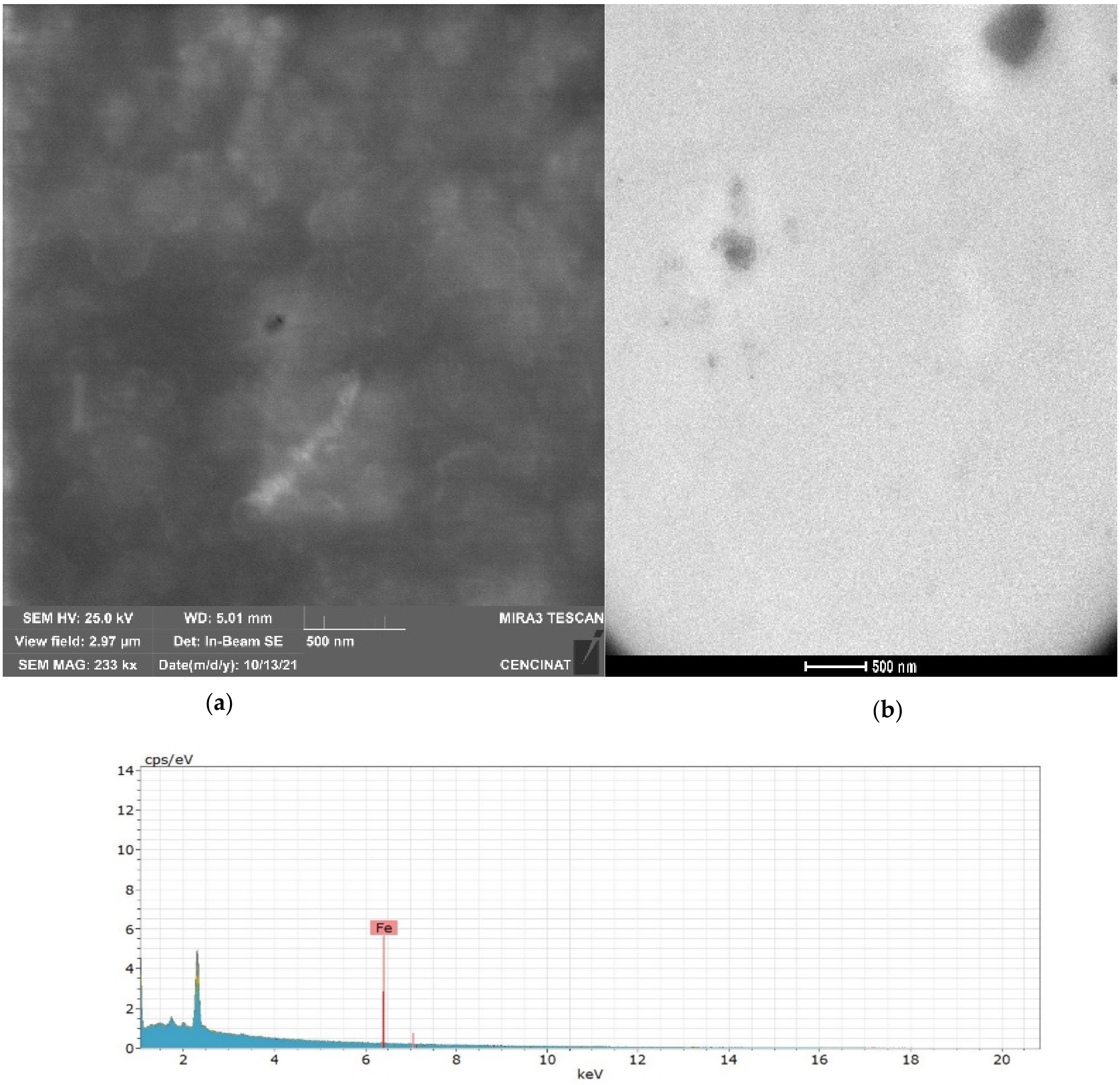

(**a**) (**b**)

(**c**)

Figure S12. (**a**) SEM, (**b**) TEM, (**c**) EDS analysis of VCL/PEGDA-MNPs-MWCNTs.

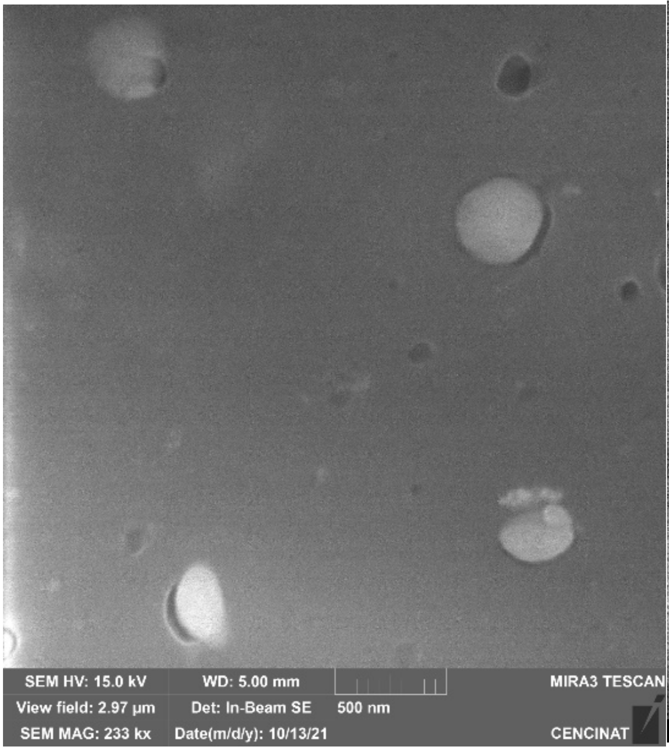

(**a**)

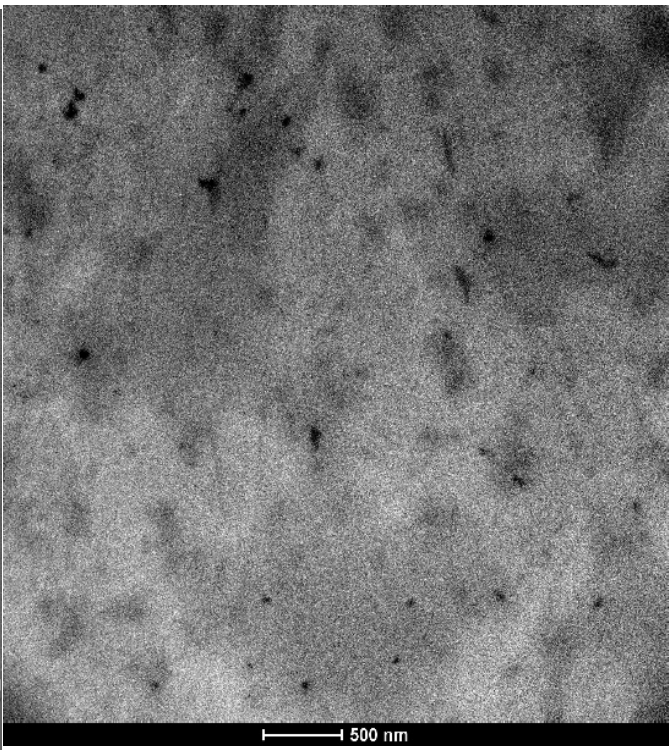

(**b**)

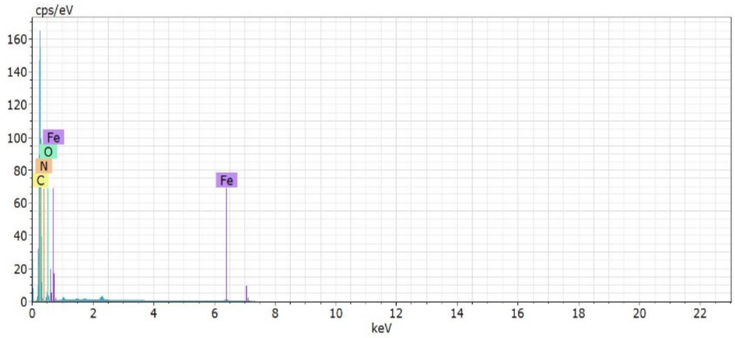

(**c**)

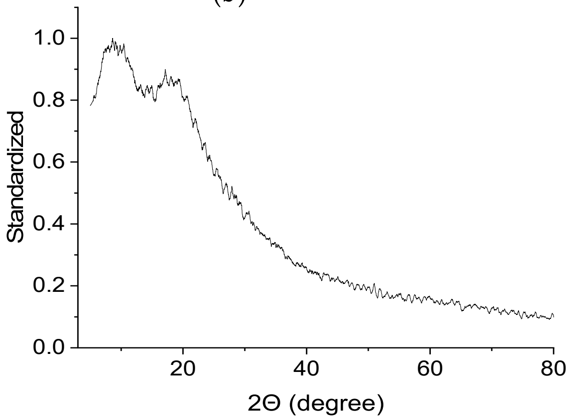

(**d**)

Figure S13. (**a**) SEM, (**b**) TEM, (**c**) EDS and (**d**) XRD analysis of VCL/PEGDA-MNPs-GO-ZnMintPc.

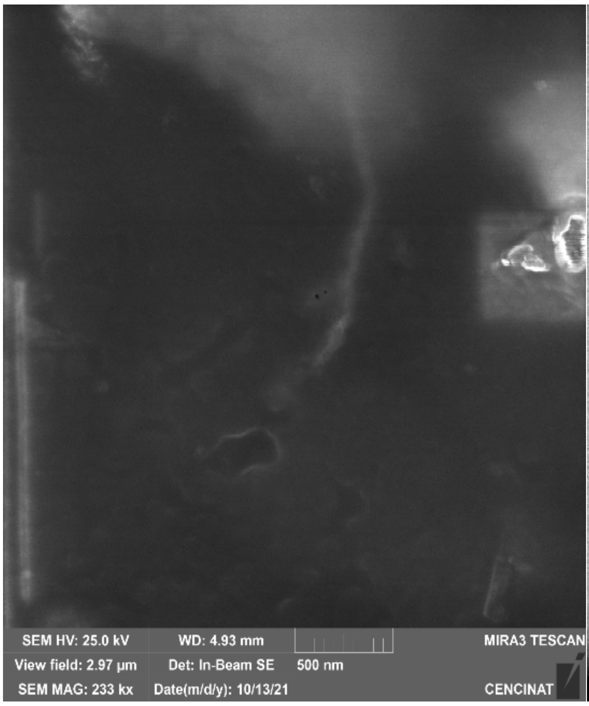

(**a**)

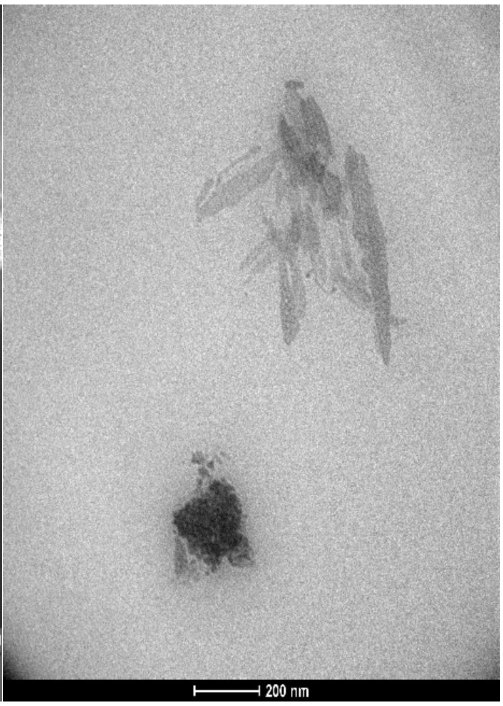

(**b**)

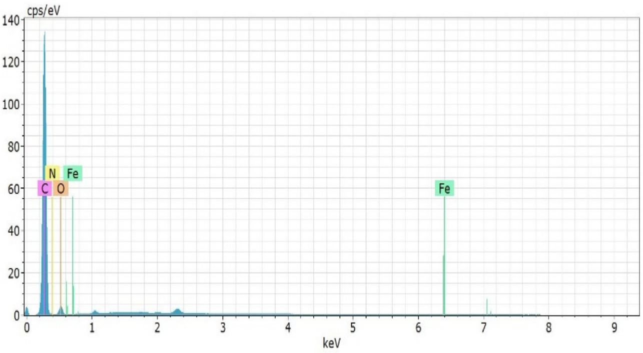

(**c**)

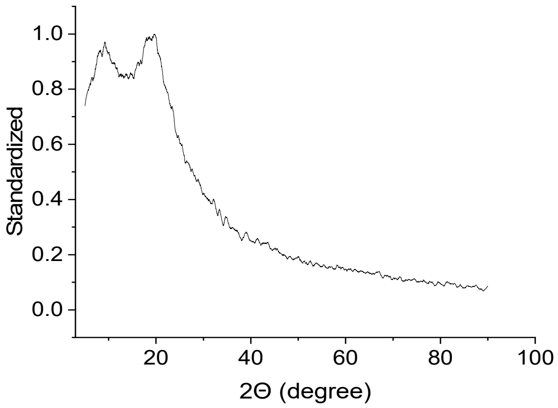

(**d**)

Figure S14. (**a**) SEM, (**b**) TEM, (**c**) EDS and (**d**) XRD analysis of VCL/PEGDA-MNPs-MWCNTs-ZnMintPc.
